# ALKBH5 promotes non-small cell lung cancer progression and susceptibility to anti-PD-L1 therapy by modulating interactions between tumor and macrophages

**DOI:** 10.1186/s13046-024-03073-0

**Published:** 2024-06-14

**Authors:** Xin Hua, Qiuli Xu, Ranpu Wu, Wei Sun, Yanli Gu, Suhua Zhu, Xin Liu, Tangfeng Lv, Yong Song

**Affiliations:** 1 Medical School of Southeast University, Nanjing, 210003 China; 2 Department of Respiratory and Critical Care Medicine, Jinling Hospital, Affiliated Hospital of Medical School, Nanjing University, Nanjing, 210002 China

**Keywords:** ALKBH5, JAK2/p-STAT3 pathway, N6-methyladenosine demethylase, Non-small cell lung cancer, Tumor-associated macrophage, Tumor microenvironment

## Abstract

**Background:**

Understanding the mechanisms that mediate the interaction between tumor and immune cells may provide therapeutic benefit to patients with cancer. The N6-methyladenosine (m6A) demethylase, ALKBH5 (alkB homolog 5), is overexpressed in non-small cell lung cancer. However, its role in the tumor microenvironment is unknown.

**Methods:**

Datasets and tissue samples were used to determine the relationship between ALKBH5 expression and immunotherapy efficacy. Bioinformatic analysis, colorimetric assay to determine m6A RNA methylation, dual luciferase reporter assay, RNA/m6A-modified RNA immunoprecipitation, RNA stability assay, and RNA sequencing were used to investigate the regulatory mechanism of ALKBH5 in non-small cell lung cancer. In vitro and in vivo assays were performed to determine the contribution of ALKBH5 to the development of non-small cell lung cancer.

**Results:**

ALKBH5 was upregulated in primary non-small cell lung cancer tissues. ALKBH5 was positively correlated with programmed death-ligand 1 expression and macrophage infiltration and was associated with immunotherapy response. JAK2 was identified as a target of ALKBH5-mediated m6A modification, which activates the JAK2/p-STAT3 pathway to promote non-small cell lung cancer progression. ALKBH5 was found to recruit programmed death-ligand 1-positive tumor-associated macrophages and promote M2 macrophage polarization by inducing the secretion of CCL2 and CXCL10. ALKBH5 and tumor-associated macrophage-secreted IL-6 showed a synergistic effect to activate the JAK2/p-STAT3 pathway in cancer cells.

**Conclusions:**

ALKBH5 promotes non-small cell lung cancer progression by regulating cancer and tumor-associated macrophage behavior through the JAK2/p-STAT3 pathway and the expression of CCL2 and CXCL10, respectively. These findings suggest that targeting ALKBH5 is a promising strategy of enhancing the anti-tumor immune response in patients with NSCLC and that identifying ALKBH5 status could facilitate prediction of clinical response to anti-PD-L1 immunotherapy.

**Supplementary Information:**

The online version contains supplementary material available at 10.1186/s13046-024-03073-0.

## Background

The high mortality rate of lung cancer, with non-small cell lung cancer (NSCLC) being the most common pathological type, remains a critical concern [1]. Established tumors have an immunosuppressive microenvironment owing to the interaction between tumor and immune cells and related signaling molecules [2, 3]. An in-depth understanding of the tumor microenvironment (TME) has driven advances in immunotherapy. Among cancer immunotherapies, immune checkpoint inhibitors, including programmed death-1 (PD-1) and programmed death-ligand 1 (PD-L1) inhibitors, are the most effective and widely used immunotherapeutic agents [4]. Although they represent a promising therapeutic approach, the resistance of cancer cells to immune checkpoint inhibitors limits their efficacy [5, 6]. Only approximately 20% of NSCLC patients achieve remission [7]. Recent research suggests that resistance to immune checkpoint inhibitors is closely related to changes in the TME [8–10]. Understanding the mechanisms that mediate the interaction between cancer cells and the TME could provide insights into immune evasion, informing the development of more effective immunotherapeutic strategies for NSCLC.

Numerous types of chemical modifications exist in cellular RNAs, including 5-methylcytosine, N6-methyladenosine (m6A), and N7-methylguanosine; such modifications increase the complexity of RNA biogenesis, structure, localization, and function [11]. Research on m6A—the most common mRNA modification—is increasing each year. The regulation of m6A involves methyl writers (METTL3/14/16 [methyltransferase-like 3/14/16] and WTAP [Wilms’ tumor 1-associated protein]), erasers (ALKBH5 [alkB homolog 5] and FTO [fat mass and obesity-related protein]), and readers (YTHDF1/2/3 [YTH domain family 1/2/3]) [12]. Better understanding of these regulatory molecules has highlighted the importance of m6A methylation in disease progression. Notably, m6A RNA modifications are reportedly involved in tumorigenesis and metastasis, and increasing evidence suggests a role for m6A RNA modifications in modulating the TME [13]. m6A RNA modification affects tumor immune escape to different degrees by reshaping the TME under hypoxic conditions, regulating acidity and metabolic reprogramming, inducing epithelial–mesenchymal transition, and influencing immune cells infiltration and immune checkpoint expression [14–16]. Immune cells are an essential component of the TME. Immune cell abnormal activation, differentiation, and expansion can cause immune failure and inhibit immune cell functions in the TME, leading to tumor escape. m6A RNA modification in immune cells is involved in the activation of T-lymphocytes, dendritic cells, and natural killer cells, as well as macrophage reprogramming [17]; thus, reshaping antitumor immunity and regulating immune escape. However, the mechanism by which m6A methylation remodels the TME and affects tumor immune monitoring has remained unknown.

In the present study, we investigated the role of ALKBH5 in regulating tumor-associated macrophages (TAMs), the phenotypes of NSCLC cells, and any interactions between the two that promoted NSCLC progression and influenced the efficacy of anti PD-L1 immunotherapy, in addition to elucidating the underlying mechanisms. Our results suggest that ALKBH5, as a biomarker for predicting anti PD-L1 immunotherapy outcomes in NSCLC patients, is a potential target for addressing anti PD-L1 immunotherapy resistance in NSCLC.

## Methods

### Reagents

Actinomycin D (M4881) was purchased from AbMole Bioscience (Houston, TX). AG490 (HY-12000) was purchased from MedChemExpress (Monmouth Junction, NJ). Matrigel matrix (356,234) was purchased from Corning (Corning, NY). Phorbol 12-myristate 13-acetate (PMA; FMS-FZ207) was purchased from Fcmacs Biotech (Nanjing, Jiangsu, China). Recombinant human CCL2 (300–04), CXCL10 (300–12), and IL-6 (200–06) were purchased from PeproTech (Cranbury, NJ). Puromycin (ST551) was purchased from Beyotime Biotechnology (Haimen, Jiangsu, China). Anti-mouse PD-L1 antibody (BE0101) was purchased from BioXcell (Lebanon, NH).

### Tissue specimens

Fresh NSCLC tissues and corresponding paracancerous tissues were collected from the Department of Thoracic Surgery of Jinling Hospital between 2020 and 2023. Paraffin-embedded NSCLC tissues were collected from the Department of Pathology of Jinling Hospital.

### Cell culture

All cell lines were obtained between 2020 and 2023. The culture medium used for human bronchial epithelial, human lung cancer and THP-1 cells was RPMI 1640 supplemented with 10% FBS (Gibco, Billings, MT). The culture medium used for Lewis lung cancer (LLC) cells and human umbilical vein endothelial cells (HUVECs) was DMEM supplemented with 10% FBS (Gibco). All cells were cultured at 37 °C with 5% CO_2_.

### Constructs and transfection

Lipofectamine 3000 (Invitrogen, Carlsbad, CA) was used to transfect cells. siRNAs targeting either ALKBH5 (si-ALKBH5) or YTHDF2 (si-YTHDF2) and a negative control siRNA (si-NC) were synthesized by RiboBio (Guangzhou, Guangdong, China). shRNA targeting ALKBH5 (sh-ALKBH5) and a negative control shRNA (sh-NC) were synthesized by Hanheng Biotechnology (Shanghai, China). Recombinant plasmids overexpressing wild-type ALKBH5 (OE-ALKBH5), the catalytic mutant ALKBH5 H204A (OE-H204A), or control (OE-NC) were constructed by Jinruisi (Nanjing, Jiangsu, China). LLC cells with stable overexpression or knockdown of ALKBH5 were infected with the lentivirus synthesized by Hanheng and selected based on puromycin (2 μg/mL) screening. The sequences used are listed in Table S1.

### Co-culture experiments

Co-culture experiments were performed using 0.4-μm Transwell chambers (Corning). The upper chamber was filled with the cancer cell suspension, and the lower chamber was filled with 100 ng/mL of PMA-stimulated THP-1 cell suspension. Following 72 h co-culture at 37 °C with 5% CO_2_, the THP-1 cells were collected.

### Cell proliferation assays

For the Cell Counting Kit-8 (CCK-8) assay, cancer cells (2,000 cells/well) were seeded in 96-well plates. CCK-8 solution (Meilunbio, Dalian, Liaoning, China) was added and incubated for 2 h before measuring the absorbance at 450 nm (BioTek, Winooski, VT). For the colony formation assay, cancer cells (1,000 cells/well) were seeded in 6-well places, cultured for 10–14 days, and then fixed and stained. Cells were counted using ImageJ (version 1.46; NIH).

### Cell migration assay

Eight-micrometer Transwell chambers (Corning) were used for cell migration assays. The cancer (4 × 10^4^ cells/well) or PMA-stimulated THP-1 (20 × 10^4^ cells/well) cell suspension was added to the upper chamber. Transfected NSCLC cells were incubated in serum-free medium for 24 h, after which the culture supernatants were collected and centrifuged at 1500 rpm for 10 min to remove the debris. The cell supernatant was used as conditioned medium. RPMI 1640 medium or the serum-free conditioned medium from the transfected NSCLC cells was supplemented with 20% FBS and added to the lower chamber. After 48 h culture, the migrated cells were fixed and stained. Cells were counted using ImageJ.

### Tube formation assay

HUVECs (1.5 × 10^4^ cells/well) were resuspended in the supernatant of NSCLC cells and seeded in Matrigel-coated 96-well plates. After 24 h incubation at 37 ºC with 5% CO_2_, tube formation was examined using an inverted microscope (MVX10; Olympus, Tokyo, Japan). Images were analyzed using ImageJ.

### RNA extraction, quantitative real-time PCR (qRT-PCR), and RNA sequencing

Total RNA was extracted using TRIzol reagent (Vazyme, Nanjing, Jiangsu, China) and reverse transcribed into cDNA. qRT-PCR was performed using a SYBR Green PCR Kit (Vazyme). GAPDH (glyceraldehyde-3-phosphate dehydrogenase) was used as an internal control. The primer sequences are listed in Table S2. RNA sequencing was performed by Novogene (Beijing, China). Differentially expressed genes and enriched pathways were identified using the “edgeR” package in R (version 3.12.1; R Foundation for Statistical Computing).

### Protein extraction, western blotting, and enzyme-linked immunosorbent assay (ELISA)

Cells and tissues were lysed in RIPA buffer (Servicebio, Wuhan, China). Cell lysates were separated by SDS-PAGE and transferred to a PVDF membrane (Millipore, Burlington, MA). The membrane was blocked and incubated with specific antibodies. Proteins were visualized using an ECL detection system (Millipore). Protein bands were quantified using ImageJ, and GAPDH was used as an internal reference for relative quantitative analysis. The antibodies used are listed in Table S3. Specific secretory proteins were detected using a human or mouse ELISA Kit (AiFang Biological, Changsha, Hunan, China), according to the manufacturer’s instructions.

### RNA stability assay

Transfected NSCLC cells were incubated with actinomycin D (5 μg/mL) for 0, 4, and 8 h. Total RNA was then extracted from each group and analyzed using qRT-PCR.

### Ribonucleoprotein immunoprecipitation (RIP) assay

The RIP assay was performed using a Magna RIP Kit (Millipore) (10% of the lysate was used as the input control). After washing and purification, RNA was analyzed using qRT-PCR. The antibodies used for RIP assay are listed in Table S3.

### Immunohistochemistry

Immunohistochemistry was performed using paraffin-embedded tissue sections. After antigen retrieval and blocking, the sections were incubated with primary and secondary antibodies and stained with DAB and hematoxylin. Immunohistochemical scores were calculated as described previously [18]. The antibodies used are listed in Table S3.

### Flow cytometry

PMA-stimulated THP-1 cells were collected after co-culture with cancer cells. The cells were incubated with specific antibodies for 30 min at 4ºC. Flow cytometry was performed using a flow cytometer (BD FACSCanto; BD, Franklin Lakes, NJ). The antibodies used are listed in Table S3.

### Immunofluorescence

Transfected NSCLC cells or PMA-stimulated THP-1 cells, after co-culture with cancer cells, were seeded on coverslips in 24-well plates. After fixation and permeabilization, the cells were incubated with primary and secondary antibodies. The cells were counterstained with DAPI and observed by fluorescence microscopy. The antibodies used are listed in Table S3.

### m6A RNA methylation

The m6A content in total RNA was quantified using an m6A RNA Methylation Assay Kit (Abcam). Total RNA (250 ng) and the m6A standard were incubated with the binding solution followed by incubation with diluted capture and detection antibodies. The m6A content was determined by measuring the absorbance at 450 nm (BioTek).

### Methylated RNA immunoprecipitation (MeRIP)–qRT-PCR

MeRIP–qRT-PCR was performed using a MeRIP m6A Kit (RiboBio). Total RNA (100 µg) was cut into 100 to 150 bp fragments. The fragmented RNA and magnetic beads coated with m6A or IgG antibodies were co-incubated for 2 h at 4 °C (10% of the total RNA was used as the input control). After elution and purification, RNA was analyzed using qRT-PCR. Potential m6A modification sites on JAK2 were predicted using the online software SRAMP (https://www.cuilab.cn/sramp) and RMBase v3.0 (https://rna.sysu.edu.cn/rmbase3/index.php). Two pairs of specific primers were designed based on the predicted modification sites. The primer sequences are listed in Table S2.

### Dual luciferase reporter assay

The wild type JAK2 sequence and the corresponding mutated sequence (replaced adenosine [A] with thymidine [T] in the potential m6A motifs) were inserted into the pmirGLO reporter vector (keyGEN BioTECH, Nanjing, Jiangsu, China) to obtain the JAK2-WT and JAK2-MUT luciferase reporter plasmids, respectively. NSCLC cells were co-transfected with the reporter plasmids and si-NC or si-ALKBH5 in 12-well plates for 48 h. Finally, using the Dual Luciferase Reporter Gene Assay kit (Yeasen Biotechnology, Shanghai, China), the cell lysate was collected, and both firefly and *Renilla* luciferase activities were detected.

### Animal experiments

C57BL/6 J mice (aged 4–6 weeks) were assigned randomly to each group. LLC cells (approximately 1 × 10^6^) with stable overexpression or knockdown of ALKBH5 were suspended in PBS, mixed with an equal volume of matrix, and injected into the axilla. When tumors reached an appropriate size, mice in the treatment group were treated with anti-PD-L1 antibody (200 µg intraperitoneally three times a week). The tumors were excised, weighed, and preserved. Tumor volume was calculated as length × width^2^ × 0.5. Tumor inhibition rate (%) was calculated using the following formula: [(tumor weight of control group—tumor weight of treatment group)/tumor weight of the control group] × 100% [19].

### Statistical analysis

Data are expressed as the mean ± SEM. Student’s *t*-test or two-way ANOVA was used to compare the differences between groups. Pearson’s correlation coefficient was determined to evaluate the correlation between two genes. All experiments were performed independently at least three times. Statistical analysis was conducted using GraphPad Prism (version 9; GraphPad Software). *P < 0.05, **P < 0.01, and ***P < 0.001 were considered statistically significant.

## Results

### ALKBH5 is upregulated in NSCLC and is associated with immune response

**Fig. 1:**
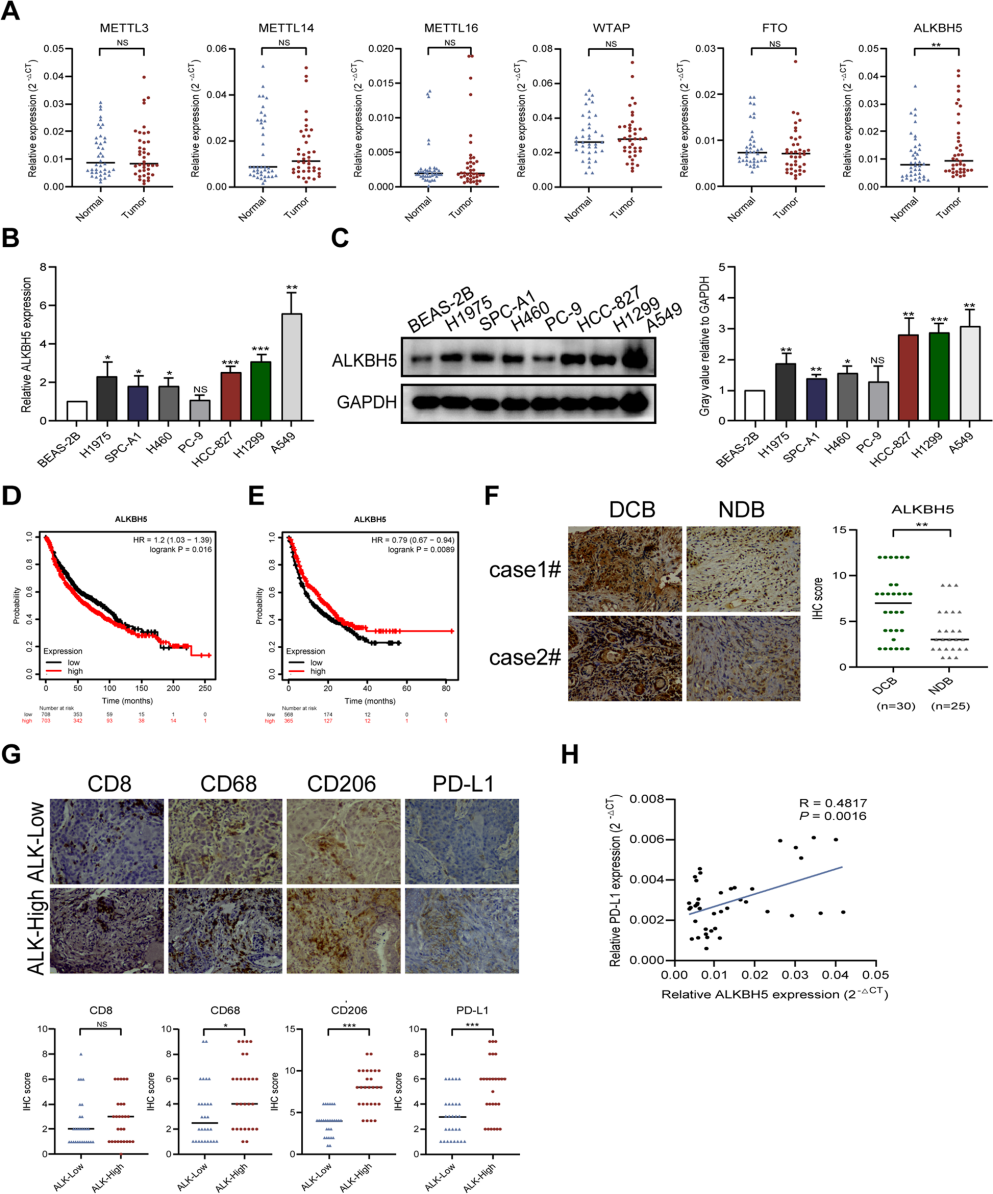
ALKBH5 is upregulated in NSCLC and is associated with the immune response.** A** qRT-PCR analysis of m6A regulators (ALKBH5, FTO, METTL3/14/16, and WTAP) in 40 pairs of NSCLC tissues and adjacent normal tissues. **B** qRT-PCR analysis of ALKBH5 expression in A549, BEAS-2B, H460, H1299, H1975, HCC-827, PC-9, and SPC-A1 cells. **C** Western blot analysis of ALKBH5 expression in A549, BEAS-2B, H460, H1299, H1975, HCC-827, PC-9, and SPC-A1 cells. GAPDH was used as the loading control. **D** Kaplan–Meier analysis of the relationship between ALKBH5 expression and overall survival in patients with lung cancer. **E** Kaplan–Meier analysis of the relationship between ALKBH5 expression and overall survival in patients with cancer treated with immunotherapy. **F** Representative immunohistochemical images of ALKBH5 in DCB (n = 30) and NDB (*n* = 25) groups (left panel; scale bar = 20 µm) and immunohistochemistry correlation analysis (right panel). **G** Representative immunohistochemical images of CD8, CD68, CD206, and PD-L1 in the low (*n* = 28) and high ALKBH5 (*n* = 27) expression groups (upper panel; scale bar = 20 µm) and immunohistochemistry correlation analysis (lower panel). **H** Pearson correlation coefficient analysis of ALKBH5 and PD-L1 expression in 40 cases of NSCLC. *P < 0.05; **P < 0.01; ***P < 0.001; NS, not significant

To study the regulation of m6A methylation in NSCLC, we analyzed the expression of methyl-modifying enzymes in 40 pairs of cancerous and paracancerous tissues. Only ALKBH5 mRNA levels were significantly upregulated in tumor tissues (Fig. 1A). We observed increased expression of ALKBH5 at both the mRNA (Fig. 1B) and protein (Fig. 1C) levels in NSCLC cells compared to BEAS-2B human bronchial epithelial cells. These results suggest that ALKBH5 may play an important role in the pathogenesis of NSCLC.

Kaplan–Meier analysis showed that increased ALKBH5 expression was associated with shorter overall survival in patients with lung cancer (Fig. 1D). Conversely, increased ALKBH5 expression was associated with longer overall survival in patients who received immunotherapy (Fig. 1E). These contradictory results suggest that ALKBH5 may play a role in modulating the TME.

To determine whether ALKBH5 expression in NSCLC tissues correlates with immunotherapy response, we collected paraffin-embedded tissue samples from 55 patients with advanced NSCLC who received immunotherapy. Patients were divided into two groups (with and without durable clinical benefit), according to the Response Evaluation Criteria in Solid Tumors (version 1.1). Statistical analysis showed that the ALKBH5 immunohistochemical score was higher in patients with durable clinical benefit (DCB) than in those with no durable clinical benefit (NDB) (Fig. 1F).

Based on ALKBH5 immunohistochemical scores, the patients were divided into high (6–12) and low (0–5) ALKBH5 subgroups. We also evaluated the relationship between ALKBH5 expression, PD-L1 (a classic immunosuppressive molecule), and immune cell markers, which play important roles in the TME. Increased ALKBH5 expression was associated with higher PD-L1, CD68, and CD206 scores (Fig. 1G). However, no correlation was observed between ALKBH5 expression and CD8 score (Fig. 1G). The positive relationship between ALKBH5 and PD-L1 expression was confirmed in 40 cases of NSCLC by performing Pearson correlation coefficient analysis (Fig. 1H). Collectively, the results show that ALKBH5 is highly expressed in NSCLC tissues and cell lines and is associated with an immunosuppressive microenvironment and immunotherapy response.

### ALKBH5 upregulates JAK2 expression to activate the JAK2/p-STAT3 pathway

**Fig. 2:**
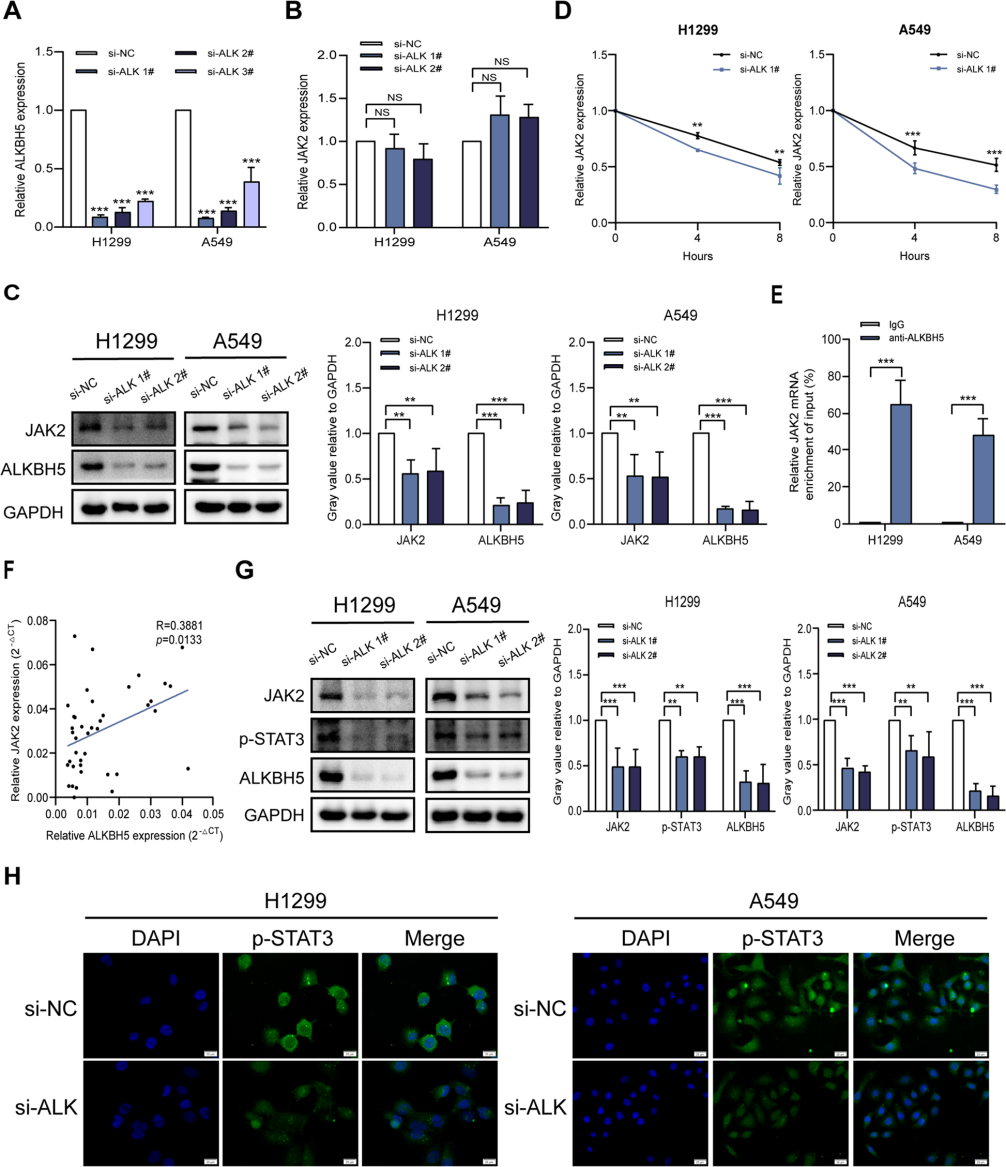
ALKBH5 upregulates JAK2 expression to activate the JAK2/p-STAT3 pathway.** A** qRT-PCR analysis of the efficiency of siRNA-mediated knockdown of ALKBH5 in A549 and H1299 cells. **B** qRT-PCR analysis of JAK2 expression in A549 and H1299 cells transfected with ALKBH5 siRNA. **C** Western blot analysis of ALKBH5 and JAK2 expression in A549 and H1299 cells transfected with ALKBH5 siRNA (loading control = GAPDH). **D** The mRNA stability of JAK2 in ALKBH5-knockdown A549 and H1299 cells treated with actinomycin D (5 µg/mL). **E** RIP–qRT-PCR analysis of the binding affinity of ALKBH5 to JAK2 in A549 and H1299 cells (control = IgG). **F** Pearson correlation coefficient analysis of ALKBH5 and JAK2 mRNA expression in 40 cases of NSCLC. **G** Western blot analysis of ALKBH5, JAK2, and p-STAT3 expression in A549 and H1299 cells with or without ALKBH5 knockdown (loading control = GAPDH). **H** Immunofluorescence analysis of p-STAT3 expression in A549 and H1299 cells with or without ALKBH5 knockdown (scale bar = 20 μm). **P < 0.01; ***P < 0.001; NS, not significant

As ALKBH5 may be involved in NSCLC pathogenesis, we investigated potential m6A-modified targets of ALKBH5 in human NSCLC cells using m6A2Target (http://m6a2target.canceromics.org) and SRAMP. We focused on JAK2 because it has multiple high-confidence predicted m6A modification sites (Figure S1) and reportedly plays an important role in modulating the TME. As JAK2 expression levels differ in different cell lines, the baseline JAK2 expression was detected in commonly used NSCLC cell lines and was found to be abundant. Similar to the expression pattern of ALKBH5, JAK2 is highly expressed in NSCLC cells compared with that in normal bronchial cells BEAS2B, with the highest expression observed in HCC-827, A549, and H1299 cells (Figure S2). To determine the relevance of ALKBH5 and JAK2, we transfected A549 and H1299 cells with siRNA to inhibit ALKBH5 expression. The transfection efficiency was confirmed by qRT-PCR (Fig. 2A). ALKBH5 knockdown inhibited the protein expression, but not mRNA expression, of JAK2 (Fig. 2B and C). However, the RNA stability assay showed a significant acceleration in the mRNA decay of JAK2 in ALKBH5-knockdown A549 and H1299 cells, indicating that ALKBH5 promotes the stability of JAK2 mRNA (Fig. 2D). As ALKBH5 promotes mRNA stability and translation, we hypothesized the presence of a negative feedback loop, which would not result in any significant change in JAK2 mRNA levels. RIP–qRT-PCR confirmed the binding of ALKBH5 to JAK2 mRNA in cancer cells (Fig. 2E). Moreover, JAK2 mRNA levels positively correlated with ALKBH5 in human NSCLC tissues (Fig. 2F). As the JAK2/p-STAT3 pathway is a classic inflammatory pathway involved in tumor progression, we subsequently explored the effects of ALKBH5 on JAK2/p-STAT3 signaling in NSCLC. Western blot analyses showed that ALKBH5 knockdown decreased JAK2 and p-STAT3 expression compared to that in control (Fig. 2G). Immunofluorescence revealed reduced p-STAT3 expression in ALKBH5-knockdown cancer cells (Fig. 2H). Collectively, these results showed that ALKBH5 upregulates JAK2 to activate the JAK2/p-STAT3 pathway in NSCLC cells.

### ALKBH5 regulates JAK2 expression in an m6A-YTHDF2-dependent manner

**Fig. 3:**
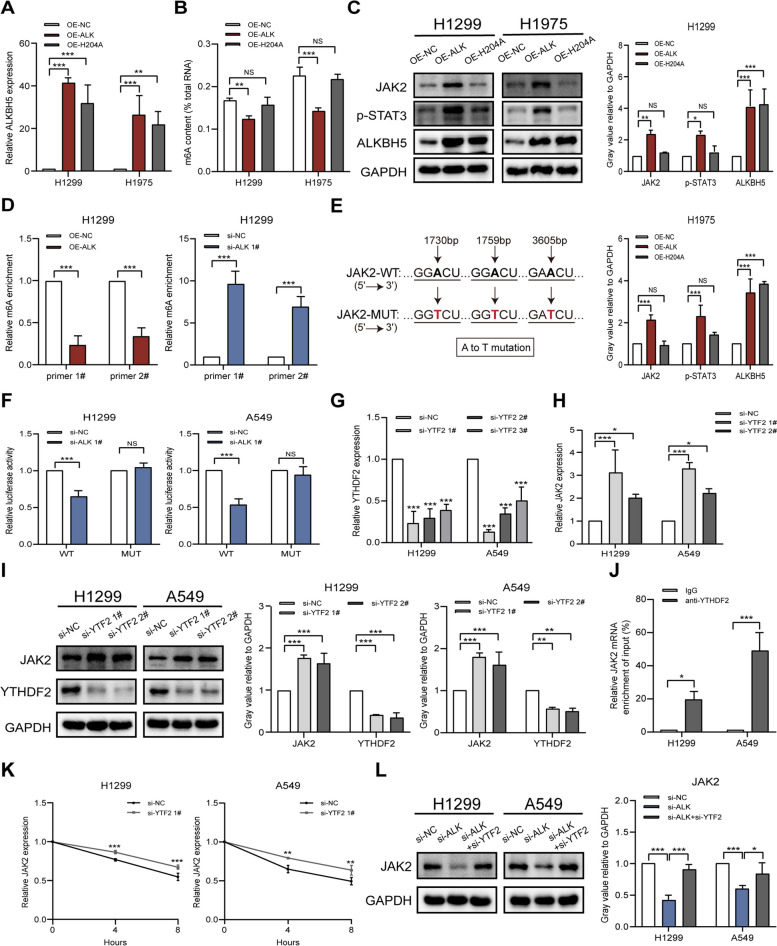
ALKBH5 regulates JAK2 expression in an m6A-YTHDF2-dependent manner.** A** qRT-PCR analysis of the efficiency of ALKBH5 overexpression in H1299 and H1975 cells transfected with OE-NC, OE-ALKBH5, and OE-H204A. **B** Overall m6A deposited RNAs in H1299 and H1975 cells transfected with OE-NC, OE-ALKBH5, and OE-H204A detected using the colorimetric m6A assay. **C** Western blot analysis of ALKBH5, JAK2, and p-STAT3 expression in H1299 and H1975 cells transfected with OE-NC, OE-ALKBH5, or OE-H204A (loading control = GAPDH). **D** MeRIP–qRT-PCR analysis of m6A modification sequences of JAK2 transcript in ALKBH5 overexpression and knockdown H1299 cells. **E** Schematic showing the sequence of the wild type and m6A motif mutation (A to T) plasmids containing luciferase reporter gene vectors. **F** Dual luciferase reporter analysis of wild type and m6A motif mutation reporter vector plasmids in A549 and H1299 cells with or without ALKBH5 knockdown. **G** qRT-PCR analysis of the efficiency of YTHDF2 knockdown in siRNA-transfected A549 and H1299 cells. **H** qRT-PCR analysis of JAK2 mRNA expression in A549 and H1299 cells transfected with YTHDF2 siRNA. **I** Western blot analysis of JAK2 and YTHDF2 expression in A549 and H1299 cells with or without YTHDF2 knockdown (loading control = GAPDH). **J** RIP–qRT-PCR analysis of the binding affinity of YTHDF2 to JAK2 in A549 and H1299 cells (control = IgG). **K** The mRNA stability of JAK2 in YTHDF2-knockdown A549 and H1299 cells treated with actinomycin D (5 µg/mL). **L** Western blot analysis of JAK2 expression in ALKBH5-knockdown A549 and H1299 cells with or without YTHDF2 deficiency (loading control = GAPDH). *P < 0.05; **P < 0.01; ***P < 0.001; NS, not significant

We constructed the OE-H204A plasmid, which showed loss of m6A demethylation activity, as described previously [20]. To determine whether ALKBH5 upregulates JAK2 via m6A modification in NSCLC, we transfected OE-H204A or OE-ALKBH5 plasmids into NSCLC cells. ALKBH5 overexpression was confirmed by qRT-PCR (Fig. 3A). m6A quantification revealed decreased m6A modification levels in cancer cells transfected with OE-ALKBH5, but not OE-H204A, compared to those transfected with OE-NC (Fig. 3B). JAK2/p-STAT3 protein expression was increased in NSCLC cells transfected with OE-ALKBH5, but not OE-H204A or OE-NC (Fig. 3C), implying that ALKBH5 regulates JAK2/p-STAT3 expression in a methyltransferase-dependent manner. The SRAMP and RMBase applications were then used to predict m6A modification sites. SRAMP predicted 7 m6A modification sites on JAK2 with very high confidence, whereas RMBase predicted 49 potential sites (Figure S3A). Three of the sites (site1: 1730 bp; site2: 1759 bp; site3: 3605 bp) were predicted by both applications (Figure S3A). For these potential m6A modification sites, two pairs of specific primers were designed to amplify fragments near the sites for the follow-up study (as the first two sites are close to each other, a combined primer pair was designed) (Figure S3B). MeRIP–qRT-PCR was then performed to determine whether ALKBH5 mediates m6A modification of JAK2 mRNA in NSCLC cells. Both m6A modification sequences on JAK2 showed different degrees of enrichment reduction in ALKBH5-overexpressing H1299 cells and show increased enrichment in ALKBH5-knockdown H1299 cells (Fig. 3D). To further confirm the role of m6A modifications in regulating JAK2 expression, we constructed potential m6A motif wild-type and mutant plasmids (for the mutants, adenine was replaced with thymidine), and inserted them into the luciferase reporter gene vectors (Fig. 3E). Dual luciferase reporter gene assay showed that in ALKBH5-knockdown NSCLC cells, the luciferase activities of the wild-type reporter were significantly reduced, whereas those of the mutant reporter were not significantly altered (Fig. 3F). This further validated the idea that ALKBH5 directly interacts with JAK2 and makes m6A modifications via these m6A sites. Overall, our results suggest that ALKBH5 upregulates JAK2 in NSCLC cells in an m6A-dependent manner.

Given that the ALKBH5 overexpression-induced decrease in m6A modification leads to JAK2 upregulation, we hypothesized that the opposite regulatory pattern may be due to the involvement of YTHDF2, which promotes mRNA degradation by recognizing m6A modifications [21]. To explore the potential relationship between YTHDF2 and JAK2, we transfected A549 and H1299 cells with siRNA to inhibit YTHDF2 expression. The transfection efficiency was confirmed by qRT-PCR (Fig. 3G). YTHDF2 knockdown increased JAK2 mRNA and protein expression (Fig. 3H and I). RIP–qRT-PCR confirmed the binding of YTHDF2 to JAK2 mRNA in NSCLC cells (Fig. 3J). The RNA stability assay showed that the decay of JAK2 mRNA was significantly slowed down in NSCLC cells subjected to YTHDF2 knockdown (Fig. 3K). Moreover, the ALKBH5 knockdown-mediated downregulation of JAK2 was reversed by silencing YTHDF2 in NSCLC cells (Fig. 3L). Thus, the ALKBH5-mediated regulation of JAK2 is mediated by the recognition of m6A modifications on JAK2 mRNA by YTHDF2.

### ALKBH5 induces PD-L1 expression and lung *cancer* cell proliferation, migration, and angiogenesis via the JAK2/p-STAT3 pathway

The JAK2/p-STAT3 pathway is an important pathway involved in the endogenous expression of PD-L1 (in tumor and immune cells) that promotes cancer progression [22–24]. The previous results show that ALKBH5 expression is positively correlated with PD-L1 expression in NSCLC tissues, and that ALKBH5 positively regulates PD-L1 expression in NSCLC cell lines (Figure S4). Therefore, we next investigated whether ALKBH5 induces PD-L1 expression and promotes NSCLC progression through the JAK2/p-STAT3 pathway.

**Fig. 4:**
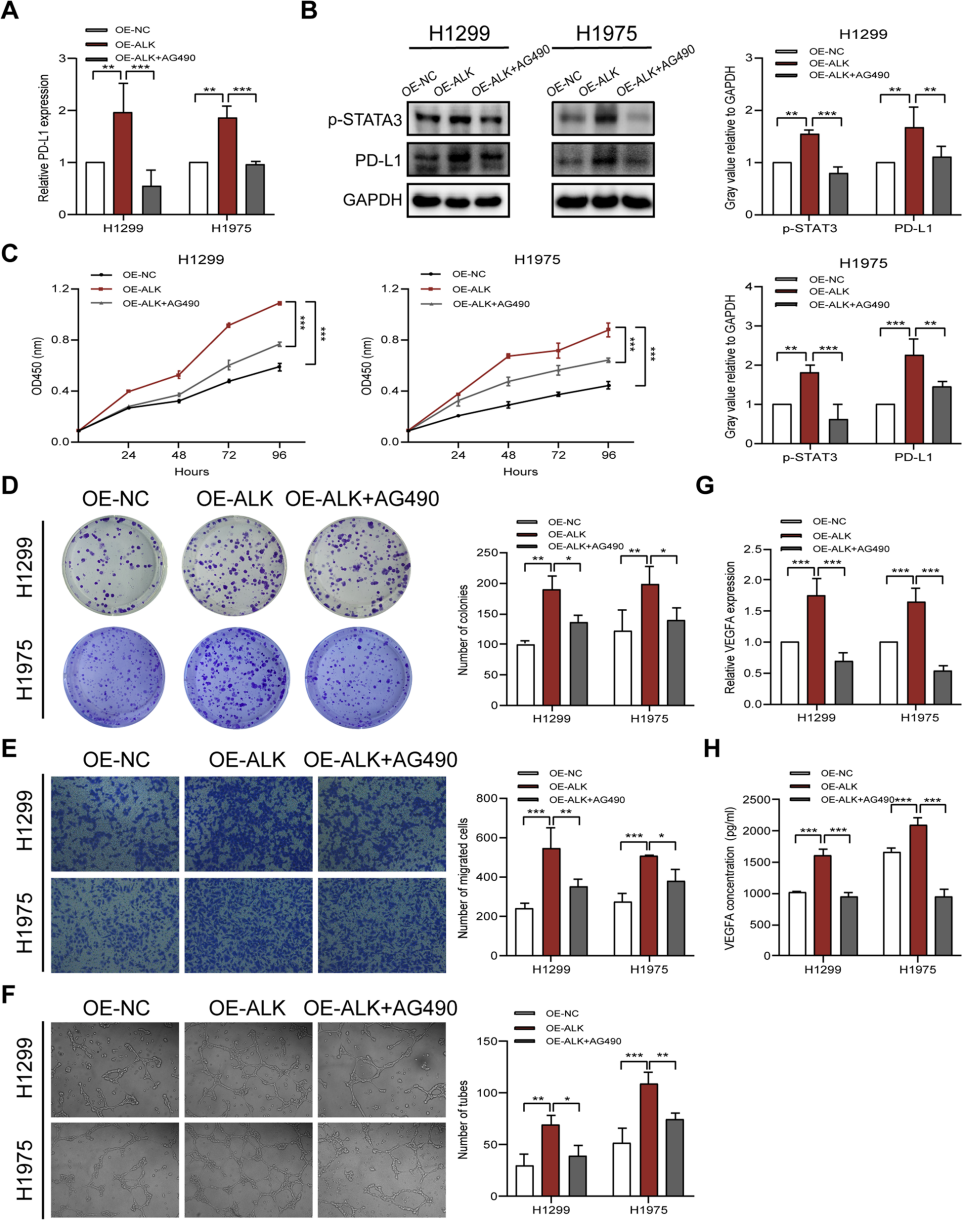
ALKBH5 induces PD-L1 expression and the malignant phenotype of NSCLC by regulating the JAK2/p-STAT3 pathway. **A** qRT-PCR analysis of PD-L1 expression in ALKBH5-overexpressing H1299 and H1975 cells treated with or without AG490 (50 μM). **B** Western blot analysis of PD-L1 and p-STAT3 expression in ALKBH5-overexpressing H1299 and H1975 cells treated with AG490 (50 μM; loading control = GAPDH). **C** CCK-8 assay of the proliferation of ALKBH5-overexpressing H1299 and H1975 cells treated with or without AG490 (50 μM). **D** Colony formation assay of ALKBH5-overexpressing H1299 and H1975 cells treated with or without AG490 (50 μM). **E** Transwell assay of the migration of ALKBH5-overexpressing H1299 and H1975 cells treated with or without AG490 (50 μM). **F** Quantification of tube formation in HUVECs treated with the supernatant of ALKBH5-overexpressing H1299 and H1975 cells treated with or without AG490 (50 μM; magnification, × 100). **G** qRT-PCR analysis of VEGFA expression in ALKBH5-overexpressing H1299 and H1975 cells treated with or without AG490 (50 μM). **H** ELISA of VEGFA secreted into the supernatant of ALKBH5-overexpressing H1299 and H1975 cells treated with or without AG490 (50 μM). *P < 0.05; **P < 0.01; ***P < 0.001

Findings of qRT-PCR and western blotting showed that PD-L1 was upregulated in H1299 and H1975 cells overexpressing ALKBH5, whereas the addition of the JAK2/p-STAT3 inhibitor, AG490, attenuated this increase (Fig. 4A and B). Subsequent CCK-8 and colony formation assays showed significant increases in the proliferative and colony-forming capacity of NSCLC cells overexpressing ALKBH5, and that AG490 reversed the effects of ALKBH5 overexpression in NSCLC cells (Fig. 4C and D). The Transwell assay findings also showed that AG490 inhibited the migration of NSCLC cells overexpressing ALKBH5 (Fig. 4E). Considering that angiogenesis is an important component of the TME, we further investigated the role of ALKBH5 in angiogenesis. The supernatant of transfected NSCLC cells and resuspended HUVECs were used to study angiogenesis. The supernatant of ALKBH5-overexpressing H1299 and H1975 cells enhanced tube formation, whereas the addition of AG490 attenuated the enhanced tube formation induced by ALKBH5 overexpression (Fig. 4F). The increase in vascular endothelial growth factor A (*VEGFA*) mRNA was also reversed by AG490 in cells overexpressing ALKBH5 (Fig. 4G). ELISA showed increased secretion of VEGFA from cells overexpressing ALKBH5, which was reversed by AG490 (Fig. 4H). Collectively, the results suggested that ALKBH5 induces PD-L1 expression and the malignant phenotype of NSCLC by regulating the JAK2/p-STAT3 pathway.

### CCL2 and CXCL10 are targets of ALKBH5

**Fig. 5:**
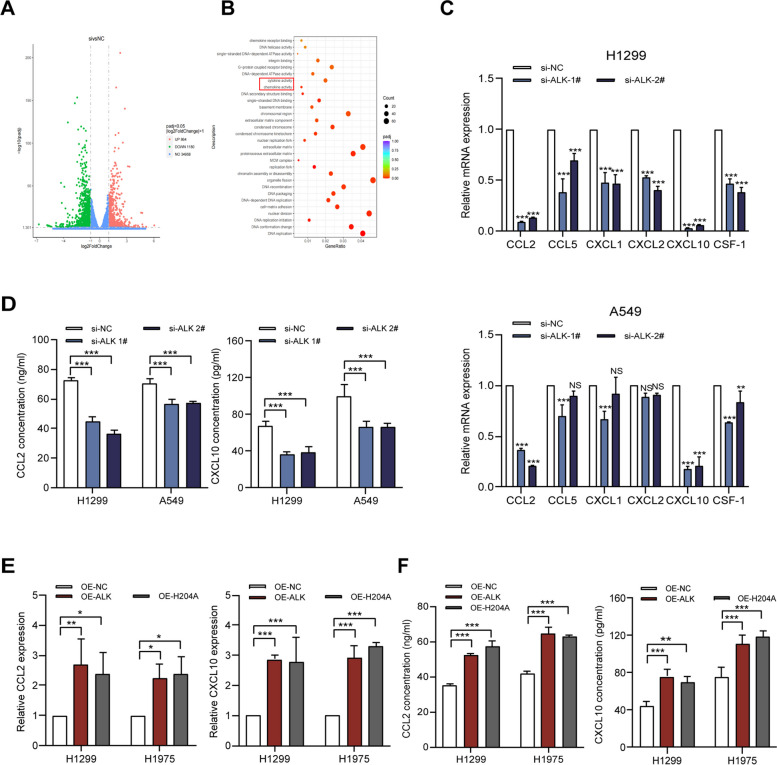
RNA sequencing identifies CCL2 and CXCL10 as targets of ALKBH5.** A** Volcano plot of up- (red) and down-regulated (green) genes (log2[Fold Change] > 1 and *P* < 0.05) in H1299 cells transfected in triplicate with si-ALKBH5 or si-NC. **B** Gene Ontology analysis of differentially expressed genes after ALKBH5 knockdown. **C** qRT-PCR analysis of the expression of six selected chemokines in A549 and H1299 cells transfected with ALKBH5 siRNA. **D** ELISA of CCL2 and CXCL10 secreted into the supernatant of ALKBH5-knockdown A549 and H1299 cells. **E** qRT-PCR analysis of CCL2 and CXCL10 expression in H1299 and H1975 cells transfected with OE-NC, OE-ALKBH5, and OE-H204A. **F** ELISA of CCL2 and CXCL10 secreted into the supernatant of H1299 and H1975 cells transfected with OE-NC, OE-ALKBH5, and OE-H204A. *P < 0.05; **P < 0.01; ***P < 0.001; NS, not significant

As lung cancer tissues with high ALKBH5 expression also showed high macrophage infiltration, we explored the mechanism by which ALKBH5 recruits macrophages to infiltrate the TME by performing RNA sequencing of NSCLC cells with or without ALKBH5 knockdown. RNA sequencing identified differentially expressed genes relative to controls. The volcano plot revealed 2,044 differentially expressed genes between these two types of cells (Fig. 5A). Gene Ontology analysis showed that ALKBH5 plays a role in regulating cytokine activity and chemokine activity (Fig. 5B). We hypothesized that ALKBH5 may mediate the recruitment of macrophages to the TME through the release of chemokines. We selected six chemokines of interest for validation and showed that CCL2 and CXCL10 mRNA levels decreased steadily in ALKBH5-knockdown A549 and H1299 cells (Fig. 5C). ELISA findings also confirmed the ALKBH5-mediated CCL2 and CXCL10 secretion by A549 and H1299 cells (Fig. 5D). It is noteworthy that the mRNA expression and secretion of CCL2 and CXCL10 were upregulated in NSCLC cells transfected with OE-ALKBH5 or OE-H204A (Fig. 5E and F). Moreover, according to SRAMP, CCL2 and CXCL10 transcripts have few high-confidence m6A modification sites (Figure S5), suggesting that ALKBH5 regulates CCL2 and CXCL10 expression in an m6A-independent manner. Collectively, the results suggest that ALKBH5 upregulates CCL2 and CXCL10 in NSCLC cells and may modulate the TME by regulating the expression of CCL2 and CXCL10.

### ALKBH5 recruits PD-L1^+^ TAMs and promotes M2 macrophage polarization by inducing CCL2 and CXCL10 secretion

**Fig. 6:**
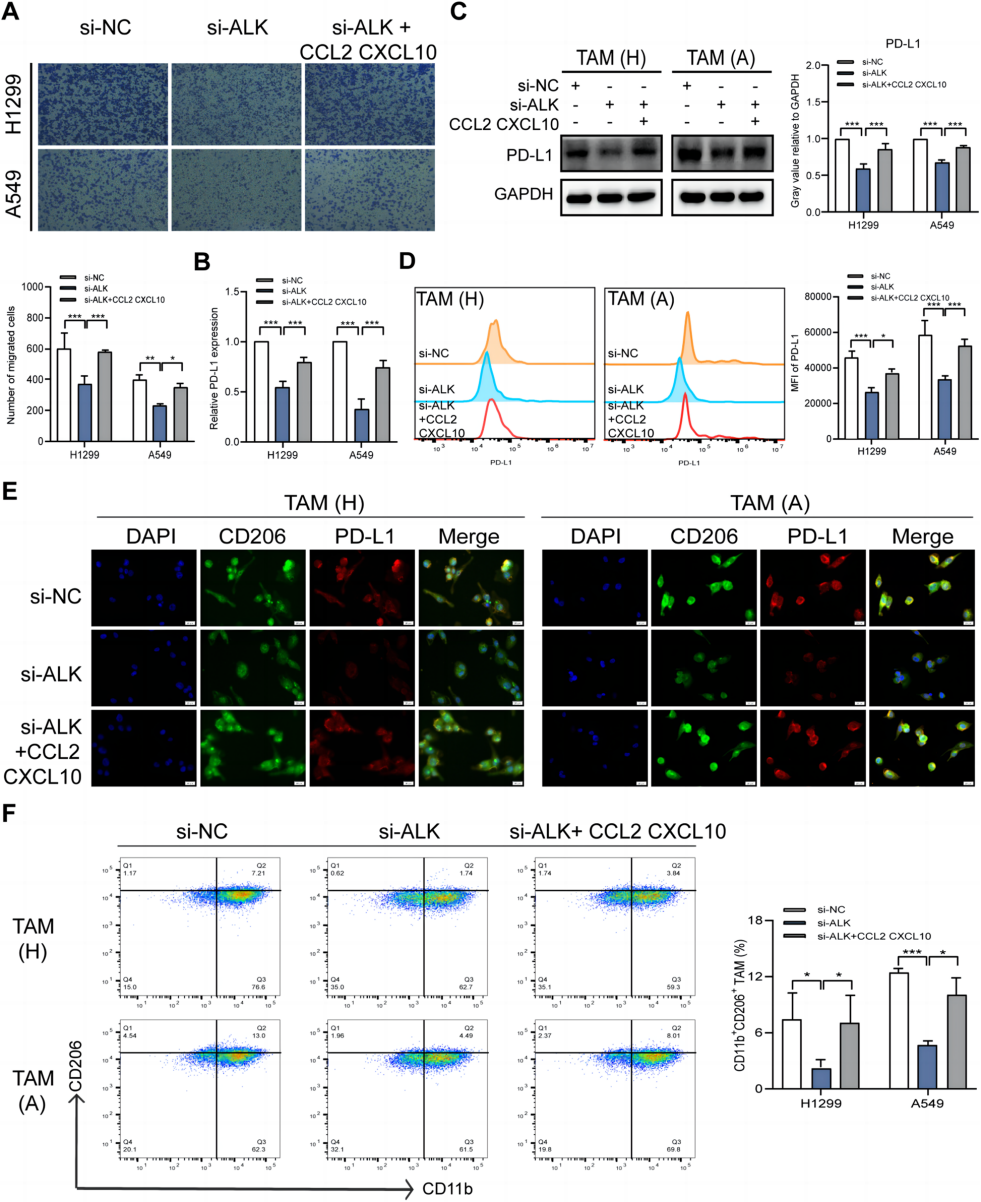
ALKBH5 recruits PD-L1^+^ TAMs and promotes M2 macrophage polarization.** A** Transwell assay of the migration of PMA-stimulated THP-1 cells co-cultured with conditioned medium from ALKBH5-knockdown A549 and H1299 cells treated with or without CCL2 (200 ng/mL) and CXCL10 (50 ng/mL) recombinant proteins. **B** qRT-PCR analysis of PD-L1 expression in PMA-stimulated THP-1 cells co-cultured with ALKBH5-knockdown A549 and H1299 cells treated with or without CCL2 (200 ng/mL) and CXCL10 (50 ng/mL) recombinant proteins. **C** Western blot analysis of PD-L1 expression in PMA-stimulated THP-1 cells co-cultured with ALKBH5-knockdown A549 and H1299 cells treated with or without CCL2 (200 ng/mL) and CXCL10 (50 ng/mL) recombinant proteins (loading control = GAPDH). **D** Flow cytometry analysis of the mean fluorescence intensity (MFI) of PD-L1 in CD206^+^ TAMs co-cultured with ALKBH5-knockdown A549 and H1299 cells treated with or without CCL2 (200 ng/mL) and CXCL10 (50 ng/mL) recombinant proteins. **E** Immunofluorescence analysis of CD206^+^PD-L1^+^ TAMs in PMA-stimulated THP-1 cells co-cultured with ALKBH5-knockdown A549 and H1299 cells treated with or without CCL2 (200 ng/mL) and CXCL10 (50 ng/mL) recombinant proteins (blue = DAPI; green = CD206; red = PD-L1; scale bar = 20 µm). **F** Flow cytometry analysis of the percentage of CD11b^+^CD206^+^ TAMs in PMA-stimulated THP-1 cells co-cultured with ALKBH5-knockdown A549 and H1299 cells treated with or without CCL2 (200 ng/mL) and CXCL10 (50 ng/mL) recombinant proteins. *P < 0.05; **P < 0.01; ***P < 0.001

CCL2 and CXCL10 are key regulators of immune cell proliferation, differentiation, and function [25–27]. To determine whether ALKBH5 recruits TAMs via CCL2 and CXCL10, we co-cultured transfected NSCLC cells and PMA-stimulated THP-1 cells. Conditioned medium from NSCLC cells with ALKBH5 knockdown inhibited macrophage migration, which was reversed by CCL2 and CXCL10 (Fig. 6A). ALKBH5 is positively correlated with PD-L1 expression in human NSCLC tissues, while macrophages and tumor cells are the main sources of PD-L1 in the TME. Therefore, we next investigated whether ALKBH5-recruited macrophages had increased PD-L1 expression. qRT-PCR and western blotting showed that CCL2 and CXCL10 reversed PD-L1 expression in TAMs, which decreased in ALKBH5-knockdown NSCLC cells (Fig. 6B and C). This finding was confirmed using flow cytometry and immunofluorescence. As most TAMs have an M2 phenotype, CD206 (an M2 marker) and PD-L1 were used to co-label macrophages and examine between-group differences in PD-L1 expression in CD206^+^ TAMs. PD-L1 expression decreased in TAMs co-cultured with ALKBH5-knockdown A549 and H1299 cells, whereas CCL2 and CXCL10 reversed this effect, as shown by the results of flow cytometry and immunofluorescence (Fig. 6D and E). Previous studies have reported that CCL2 and CXCL10 play a regulatory role in macrophage polarization; therefore, we collected the co-cultured macrophages to evaluate the expression of polarization markers. Flow cytometry revealed a lower percentage of CD11b^+^CD206^+^ M2 macrophages in co-cultures of ALKBH5-knockdown A549 and H1299 cells, which was reversed by CCL2 and CXCL10 (Fig. 6F). The decrease in the expression of M2 markers (CD163, CD206, and IL-10) in macrophages co-cultured with ALKBH5-knockdown A549 and H1299 cells was accompanied by an increase in the expression of M1 markers (CD86, IL-1β, and TNF-α), which was reversed by CCL2 and CXCL10 (Figure S6). Collectively, the results suggest that ALKBH5 recruits PD-L1^+^ TAMs and promotes M2 macrophage polarization by inducing the secretion of CCL2 and CXCL10.

### TAM-secreted IL-6 and ALKBH5 synergistically to activate the JAK2/p-STAT3 pathway

**Fig. 7:**
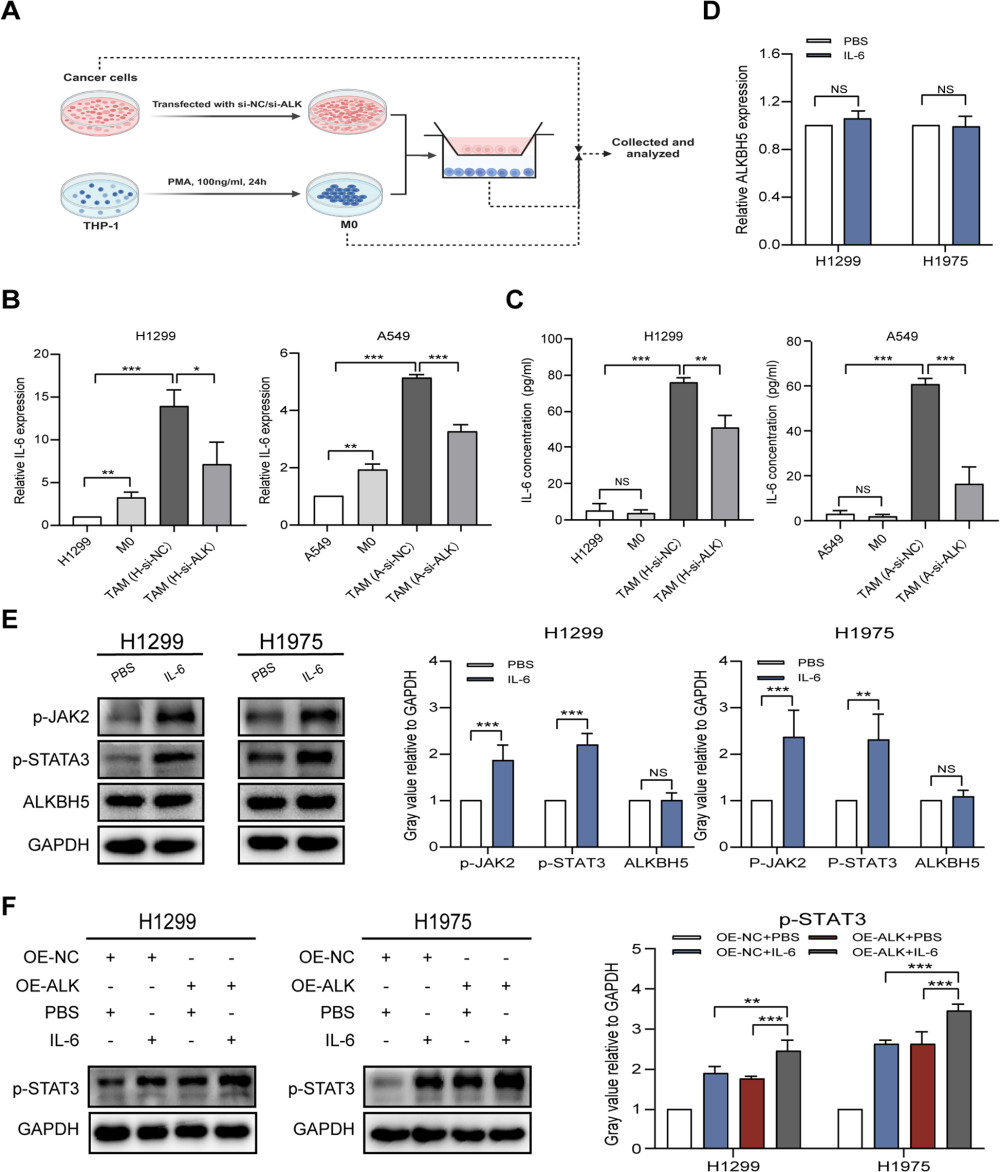
ALKBH5 has a synergistic effect with IL-6 on the activation of the JAK2/p-STAT3 pathway.** A** Schematic of the co-culture model (created with BioRender.com). **B** qRT-PCR analysis of IL-6 expression in A549 or H1299 cells, THP-1 macrophages, and PMA-stimulated THP-1 cells co-cultured with ALKBH5-knockdown or control A549 and H1299 cells. **C** ELISA of IL-6 secreted from A549 or H1299 cells, THP-1 macrophages, and PMA-stimulated THP-1 cells co-cultured with ALKBH5-knockdown or control A549 and H1299 cells. **D** qRT-PCR analysis of ALKBH5 expression in H1299 and H1975 cells treated with or without IL-6 (20 ng/mL). **E** Western blot analysis of ALKBH5, p-JAK2, and p-STAT3 expression in H1299 and H1975 cells treated with or without IL-6 (20 ng/mL; loading control = GAPDH). **F** Western blot analysis of p-STAT3 expression in ALKBH5-overexpressing H1299 and H1975 cells treated with or without IL-6 (20 ng/mL; loading control = GAPDH). *P < 0.05; **P < 0.01; ***P < 0.001; NS, not significant

TAMs are an important source of IL-6 [28], which regulates the expression of m6A regulators and activates the JAK2/p-STAT3 pathway in cancer cells [29, 30]. Therefore, we investigated the role of IL-6 in this model. First, we measured IL-6 expression in NSCLC cells, THP-1 macrophages, and TAMs (Fig. 7A). We observed significant upregulation of IL-6 in TAMs (compared with THP-1 macrophages and NSCLC cells) (Fig. 7B). IL-6 mRNA expression was decreased in TAMs co-cultured with ALKBH5-knockdown NSCLC cells compared to control (Fig. 7B). Similarly, IL-6 secretion increased in TAM supernatants and decreased in NSCLC cell and THP-1 macrophage supernatants (Fig. 7C). IL-6 secretion decreased in TAM supernatants co-cultured with NSCLC cells transfected with si-ALKBH5 compared to those transfected with si-NC (Fig. 7C). This suggested that TAMs are the main source of IL-6 in the TME, and that TAMs co-cultured with NSCLC cells highly expressing ALKBH5 also secrete more IL-6. Next, we explored whether IL-6 in the TME regulates ALKBH5 expression in NSCLC cells. We found that IL-6 did not increase the mRNA expression of ALKBH5 in NSCLC cells (Fig. 7D). In addition, exogenous IL-6 activated the p-JAK2/p-STAT3 pathway but did not increase the protein expression of ALKBH5 in NSCLC cells, suggesting that IL-6 has no direct regulatory effect on ALKBH5 (Fig. 7E). Moreover, ALKBH5 overexpression had a synergistic effect with IL-6 on the activation of downstream p-STAT3 signaling (Fig. 7F). Collectively, these results show that ALKBH5 recruits TAMs via CCL2 and CXCL10. TAMs induce IL-6 secretion, which exerts a synergistic effect along with ALKBH5 to activate p-STAT3 signaling in NSCLC cells, thus maintaining an immunosuppressive microenvironment that drives tumorigenesis.

### ALKBH5 promotes tumor growth in vivo and is sensitive to anti PD-L1 therapy

**Fig. 8:**
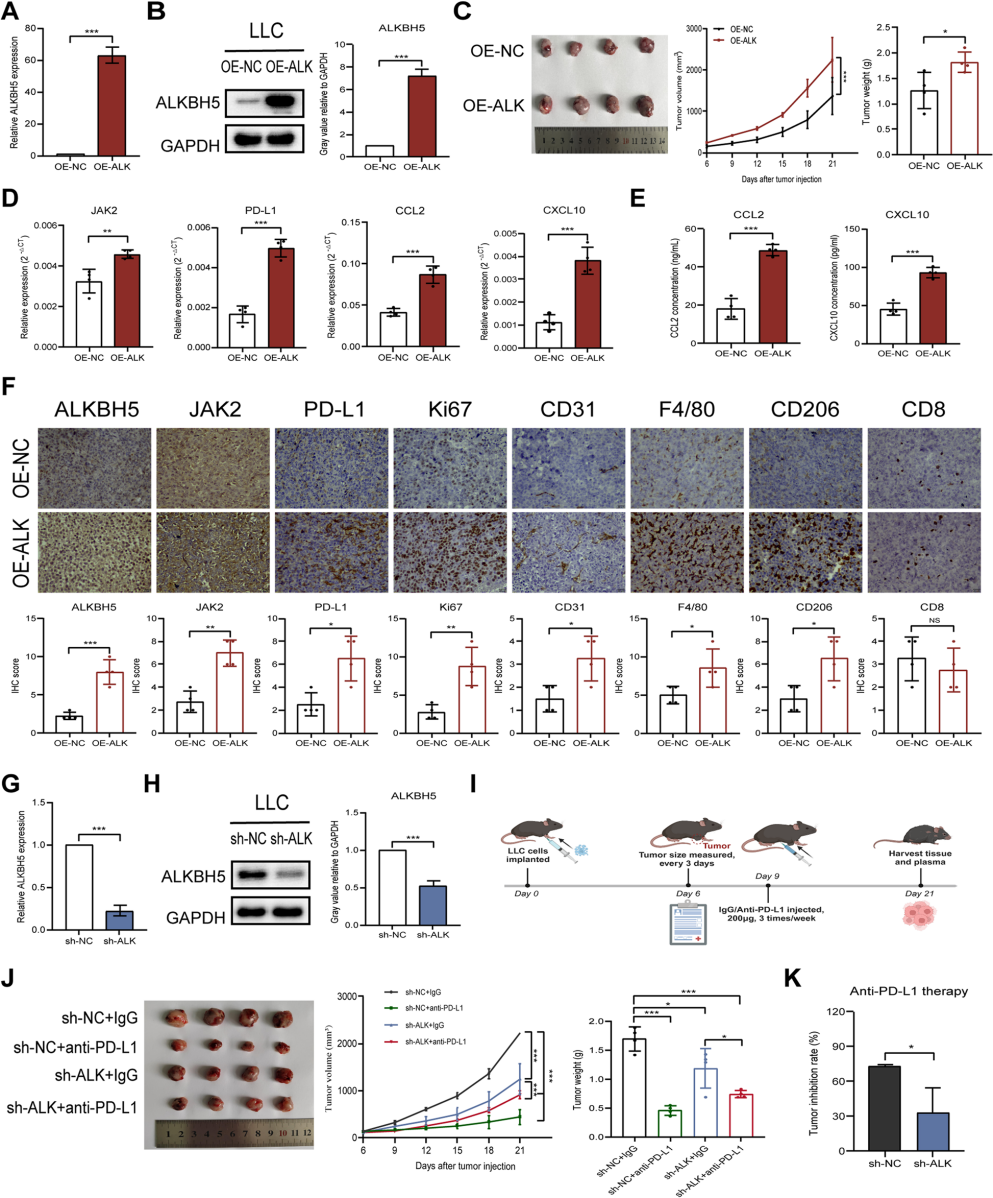
ALKBH5 promotes tumor growth in vivo and is sensitive to anti PD-L1 therapy.** A** qRT-PCR analysis of the efficiency of ALKBH5 overexpression in LLC cells. **B** Western blot analysis of the efficiency of ALKBH5 overexpression in LLC cells (loading control = GAPDH). **C** Left panel: ALKBH5 overexpression promotes tumor growth in C57BL/6 J mice (*n* = 4). Middle panel: Tumor volumes measured by growth curve every three days from day 6 to day 21 after cell transplantation. Right panel: Tumor weight determined immediately after removal from C57BL/6 J mice. **D** qRT-PCR analysis of tumor mRNA expression of CCL2, CXCL10, JAK2, and PD-L1. **E** ELISA of CCL2 and CXCL10 in plasma. **F** Representative immunohistochemical images of ALKBH5, CD8, CD31, CD206, F4/80, JAK2, Ki-67, and PD-L1 expression in ALKBH5-overexpressing and control mice (upper panel; scale bar = 20 µm) and immunohistochemistry correlation analysis (lower panel). **G** qRT-PCR analysis of the efficiency of ALKBH5 knockdown in LLC cells. **H** Western blot analysis of the efficiency of ALKBH5 knockdown in LLC cells (loading control = GAPDH). **I** Schematic of the cell transplantation and drug administration experiment (created with BioRender.com). **J** Left panel: ALKBH5 knockdown and anti-PD-L1 therapy attenuated tumor growth in C57BL/6 J mice (*n* = 4). Middle panel: Tumor volumes measured by growth curve every three days from days 6 to 21 after cell transplantation. Right panel: Tumor weight determined immediately after removal from C57BL/6 J mice. **K** Tumor inhibition rate of ALKBH5-knockdown and control mice treated with anti-PD-L1 therapy. *P < 0.05; **P < 0.01; ***P < 0.001; NS, not significant

To clarify the role of ALKBH5 in controlling tumor growth and sensitivity to immunotherapy, we established an in vivo subcutaneous xenograft model. LLC cell lines stably overexpressing ALKBH5 were implanted subcutaneously into mice (Fig. 8A and B). The tumor burden was higher in ALKBH5-overexpressing mice than in wild-type mice (Fig. 8C). Analysis of tumor-associated RNA revealed high JAK2, PD-L1, CCL2, and CXCL10 expression in ALKBH5-overexpressing mice compared to wild-type mice (Fig. 8D). Similar trends were observed in the plasma levels of secreted CCL2 and CXCL10 (Fig. 8E). Immunohistochemistry showed increased expression of ALKBH5, CD31, CD206, F4/80, JAK2, Ki-67, and PD-L1, but not CD8, in ALKBH5-overexpressing mice compared to wild-type mice (Fig. 8F).

To clarify the relationship between ALKBH5 expression and immunotherapy response, ALKBH5-knockdown LLC cells were used to establish a subcutaneous mouse model of NSCLC. The knockdown efficiency was confirmed by qRT-PCR and western blotting (Fig. 8G and H). The mice were divided into four groups according to ALKBH5 expression and anti-PD-L1 treatment. The process of drug administration experiment was shown in Fig. 8I. The results showed that ALKBH5 knockdown and anti-PD-L1 treatment inhibited tumor growth and reduced tumor burden (Fig. 8J). The inhibitory effect of anti-PD-L1 therapy on tumor growth was attenuated in ALKBH5-knockdown mice, suggesting that lung cancer cells with high ALKBH5 expression are more sensitive to anti-PD-L1 therapy (Fig. 8K). Collectively, the in vivo studies showed that ALKBH5 promotes cancer progression by facilitating tumor growth and maintaining an immunosuppressive microenvironment, whereas anti-PD-L1 therapy is more effective in tumors with high ALKBH5 expression.

## Discussion

Immune cells, including macrophages, granulocytes, lymphocytes, and dendritic cells can create a microenvironment that regulates tumor progression [31–33]. Additionally, tumor cells can recruit immune cells, or affect the activity of immune cells, by secreting various inflammatory cytokines and chemokines [34–36]. This interaction maintains an immunosuppressive microenvironment. TAMs (an important component of the TME) have an M2-like phenotype, as well as immunosuppressive and pro-tumor functions [37]. Examining the interaction between tumor cells and TAMs and understanding the drivers of this interaction may provide novel avenues for NSCLC treatment.

PD-L1 upregulation is an important mechanism of tumor immune escape. Tumor cell-expressed PD-L1 binds to the PD-1 receptor on CD8^+^ T lymphocytes, thereby inhibiting the activity of effector T cells, which weakens the anti-tumor activity of the immune system [38]. TAMs are an important source of PD-L1 in the TME. On the one hand, PD-L1 expression in TAMs is induced by cytokines and chemokines, while on the other hand, it is regulated by intrinsic carcinogenic pathways, with the former playing a more important role [39, 40]. However, the relationship between PD-L1^+^ TAMs and tumor progression remains controversial, and the relevance of PD-L1^+^ TAMs to immunotherapy outcomes is unclear. Most studies [39–41] support the role of PD-L1^+^ TAMs in negatively regulating T cell activity and promoting immune escape. However, some studies [42, 43] also suggest that PD-L1^+^ TAMs may contribute to immune activation in the TME, which is beneficial to the prognosis of patients with cancer.

According to previous studies, m6A modification plays an important role in regulating PD-L1 expression. ALKBH5, FTO, METTL3, and METTL16 directly modify PD-L1 mRNA by m6A methylation [44–48]. ALKBH5 may also indirectly regulate PD-L1 expression via the m6A modification of other mRNAs in cancer cells [16, 49]. In addition, ALKBH5 can promote PD-L1 expression in TAMs in liver cancer [50]. However, few studies have looked into the regulatory role of m6A methylation in PD-L1 expression and immune surveillance in lung cancer. Yu et al. found that METTL3 directly affects the stability of PD-L1 mRNA and the efficacy of anti-PD-1 in an m6A-dependent manner [51]. Liu et al. and Sun et al. found that METTL3 regulates the ubiquitination-mediated degradation of PD-L1 protein via methylation modification of some non-coding RNAs, thus affecting the infiltration and activation of CD8^+^T cells [52, 53]. In addition, Xu et al. showed that the low expression of the METTL3/IL-18 axis in cancer-associated fibroblasts promotes PD-L1 expression in NSCLC cells, thus inhibiting the immune activity of CD8^+^T and aggravating the PD-L1 mediated immunosuppression of NSCLC [54]. Based on this information, we explored the effect of the demethylating enzyme ALKBH5 on the anti-PD-L1 efficacy in NSCLC through the cross-talk of tumor cells and TAMs, hoping to shed further light on the role of m6A modification in immunotherapy. We found, on the one hand, that ALKBH5 regulates JAK2 expression through m6A methylation, activating the JAK2/p-STAT3 pathway to increase PD-L1 expression and promote NSCLC progression. On the other hand, CCL2 and CXCL10 are transcriptionally activated by ALKBH5, further recruiting PD-L1^+^ TAMs and promoting M2 macrophage polarization. Increased ALKBH5 expression confers increased sensitivity to anti-PD-L1 therapy in lung cancer cells. This conclusion is also supported by Liu et al. [55], who found that among nearly 500 patients with NSCLC, those harboring macrophages expressing PD-L1 had better overall survival when treated with anti-PD-1/PD-L1 therapy.

Herein, we also showed that ALKBH5-recruited TAMs secrete IL-6 into the TME of NSCLC cells. IL-6 activated the JAK2/p-STAT3 pathway in NSCLC cells, which exerts a synergistic effect with ALKBH5, thus maintaining an immunosuppressive microenvironment that drives tumorigenesis. These findings further our understanding of the interaction between tumor cells and TAMs in the TME.

**Fig. 9:**
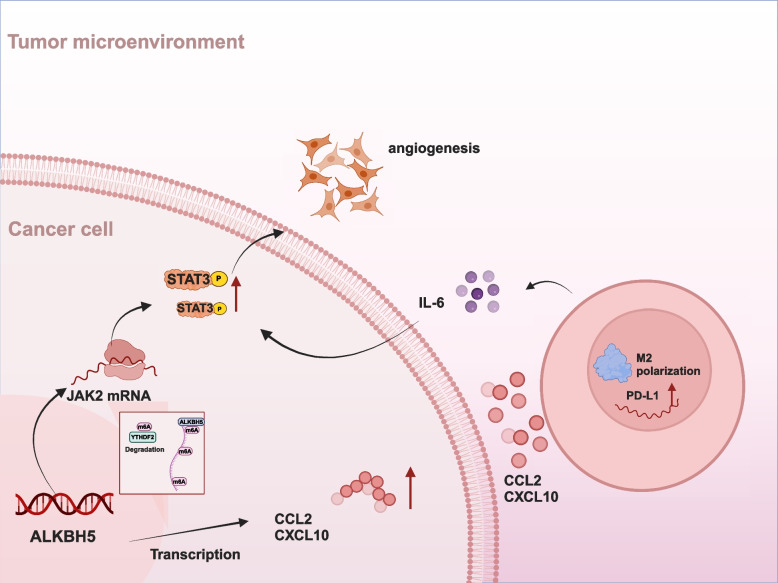
Illustrative model of the proposed mechanism.ALKBH5 promotes NSCLC progression and susceptibility to anti-PD-L1 therapy by modulating the interactions between tumor and macrophages (created with BioRender.com)

ALKBH5/JAK2/p-STAT3 and ALKBH5/CCL2/CXCL10 expression is involved in the development of a malignant phenotype and immunosuppressive microenvironment. This promotes the malignant behavior of NSCLC cells, increases the infiltration of TAMs, and upregulates PD-L1 expression in NSCLC cells and TAMs. The dual modulatory effect of ALKBH5 on PD-L1 expression in the TME, along with the synergistic effect of ALKBH5 and IL-6, may improve the efficacy of anti PD-L1 therapy (Fig. 9). However, this study has several limitations. First, we only focused on the role of ALKBH5. The synergistic or antagonistic effects of other m6A regulators on ALKBH5 have not been explored. Second, ALKBH5 induces PD-L1 expression in TAMs; however, the pathways that are activated during this process have not been investigated.

## Conclusions

The findings of the present study imply that ALKBH5 is a potential biomarker to predict the response of patients with NSCLC to anti PD-L1 immunotherapy, and that targeting ALKBH5 may be a promising strategy to enhance anti-tumor immunity. They also contribute to our understanding of the mechanism underlying m6A modifications in the TME of NSCLC. Nevertheless, further studies are needed to target m6A regulators to enhance both the anti-tumor immune response and the efficacy of combining targeted m6A modification with immunotherapy.

### Supplementary Information

Additional file 1: Table S1. siRNA sequences.

Additional file 2: Table S2. qRT-PCR primer sequences.

Additional file 3: Table S3. Antibody sources and dilutions.

Additional file 4: Figure S1. SRAMP predicted m6A modification sites on JAK2. A m6A modification sites predicted on the main JAK2 transcript based on online software SRAMP.

Additional file 5: Figure S2. Basal expression level of JAK2 in normal bronchial epithelial cells and common NSCLC cells. A qRT-PCR analysis of JAK2 expression in A549, BEAS-2B, H460, H1299, H1975, HCC-827, PC-9, and SPC-A1 cells. B Western blot analysis of JAK2 expression in A549, BEAS-2B, H460, H1299, H1975, HCC-827, PC-9, and SPC-A1 cells. GAPDH was used as the loading control. *P < 0.05; **P < 0.01; ***P < 0.001.

Additional file 6: Figure S3. Construction of specific primers based on potential m6A modification sites on JAK2 transcript. A The online software SRAMP and RMBase V3.0 predicted 7 and 49 potential m6A modification sites on JAK2 transcript, respectively. B Three overlapping potential m6A modification sites on the JAK2 transcript were located at 1730 bp, 1759 bp, and 3605 bp on the JAK2 transcript from the 5’ end. Two pairs of specific primers were constructed to amplify the fragments near the sites (sequence 1 and sequence 2).

Additional file 7: Figure S4. ALKBH5 induces PD-L1 expression in NSCLC cells. A qRT-PCR analysis of PD-L1 expression in A549 and H1299 cells transfected with ALKBH5 siRNA. B Western blot analysis of PD-L1 expression in A549 and H1299 cells transfected with ALKBH5 siRNA (loading control = GAPDH). **P < 0.01; ***P < 0.001.

Additional file 8: Figure S5. SRAMP predicted m6A modification sites on CCL2 and CXCL10. A m6A modification sites predicted on CCL2 based on online software SRAMP. B m6A modification sites predicted on CXCL10 based on online software SRAMP.

Additional file 9: Figure S6 ALKBH5 promotes M2 macrophage polarization in NSCLC. A qRT-PCR analysis of the expression of M1 (CD86, IL-1β, and TNF-α) and M2 (CD163, CD206, and IL-10) polarization markers in PMA-stimulated THP-1 cells co-cultured with ALKBH5-knockdown H1299 cells treated with or without CCL2 (200 ng/mL) and CXCL10 (50 ng/mL) recombinant proteins. B qRT-PCR analysis of the expression of M1 (CD86, IL-1β, and TNF-α) and M2 (CD163, CD206, and IL-10) polarization markers in PMA-stimulated THP-1 cells co-cultured with ALKBH5-knockdown A549 cells treated with or without CCL2 (200 ng/mL) and CXCL10 (50 ng/mL) recombinant proteins. *P < 0.05; **P < 0.01; ***P < 0.001; NS, not significant.

## Data Availability

The datasets used and/or analyzed during the current study are available from the corresponding author on reasonable request.

## Abbreviations

ALKBH5: AlkB homolog 5
CCK-8: Cell Counting Kit-8
DCB: Durable clinical benefit
ELISA: Enzyme-linked immunosorbent assay
FTO: Fat mass and obesity-related protein
GAPDH: Glyceraldehyde-3-phosphate dehydrogenase
HUVEC: Human umbilical vein endothelial cell
LLC: Lewis lung cancer
m6A: N6-methyladenosine
MeRIP: Methylated RNA immunoprecipitation
METTL: Methyltransferase-like
NC: Negative control
NDB: No durable clinical benefit
NSCLC: Non-small cell lung cancer
PD-1: Programmed death-1
PD-L1: Programmed death-ligand 1
PMA: Phorbol 12-myristate 13-acetate
qRT-PCR: Quantitative real-time PCR
RIP: Ribonucleoprotein immunoprecipitation
TAM: Tumor-associated macrophage
TME: Tumor microenvironment
VEGFA: Vascular endothelial growth factor A
WTAP: Wilms’ tumor 1-binding protein
YTHDF: YTH domain family
