# Supplementary material for: ALKBH5 promotes non-small cell lung cancer progression and susceptibility to anti-PD-L1 therapy by modulating interactions between tumor and macrophages
Source: J Exp Clin Cancer Res. 2024 Jun 14;43:164. doi: 10.1186/s13046-024-03073-0 (PMC11177518; doi:10.1186/s13046-024-03073-0)
Supplement: Supplementary file 1 — Additional file 1: Table S1. siRNA sequences. [file 13046_2024_3073_MOESM1_ESM.doc]

**Table S1 siRNA sequences (5'–3')**

| **Target** | **Sequence (5'–3')** |
| --- | --- |
| si-ALKBH5#1 | GCUGCAAGUUCCAGUUCAATT |
| si-ALKBH5#2 | GGGCCAAGCGCAAGUAUCATT |
| si-ALKBH5#3 | ACAAGTACTTCTTCGGCGA |
| si-YTHDF2#1 | CAAGGAAACAAAGTGCAAA |
| si-YTHDF2#2 | CTGCCATGTCAGATTCCTA |
| si-YTHDF2#3 | AAGGACGUUCCCAAUAGCCAATT |
