# Supplementary material for: ALKBH5 promotes non-small cell lung cancer progression and susceptibility to anti-PD-L1 therapy by modulating interactions between tumor and macrophages
Source: J Exp Clin Cancer Res. 2024 Jun 14;43:164. doi: 10.1186/s13046-024-03073-0 (PMC11177518; doi:10.1186/s13046-024-03073-0)
Supplement: Supplementary file 2 — Additional file 2: Table S2. qRT-PCR primer sequences. [file 13046_2024_3073_MOESM2_ESM.doc]

**Table S2 qRT-PCR primer sequences (5'–3')**

| **Gene** | **Sequence (5'–3')-Forward** | **Sequence (5'–3')-Reverse** |
| --- | --- | --- |
| Human METTL3 | gctgaccattccaagctctc | atttcttggctggctccttt |
| Human METTL14 | tcgtaagctcccggtgaa | tttaacacggcaccaatgc |
| Human METTL16 | tgagaggtggctgttggtc | agtgagctaagatcgcacca |
| Human WTAP | gcgactagcaaccaaggaac | gcgtaaacttccaggcactc |
| Human FTO | gagagcgcgaagctaagaaa | aaccaggtcccgaaataagc |
| Human ALKBH5 | ttcaagcctattcgggtgtc | tgaagcatagctgggtggta |
| Human PD-L1 | cctggagggagaccttgata | caaattgaggcattgagtgg |
| Human JAK2 | gtcatggcccaatttcgat | ttctttgtcccactgaggtt |
| Human YTHDF2 | acgttcccaatagccaactg | ttcctcttggcgtttctcat |
| Human VEGFA | cccactgaggagtccaacat | tccctttcctcgaactgatt |
| Human CCL2 | tctgtgcctgctgctcatag | catggaatcctgaacccact |
| Human CCL5 | cgctgtcatcctcattgcta | ccagacttgctgtccctctc |
| Human CXCL1 | CAAACCGAAGTCATAGCCACA | CTCCTAAGCGATGCTCAAACA |
| Human CXCL2 | GGCAGAAAGCTTGTCTCAACCC | CTCCTTCAGGAACAGCCACCAA |
| Human CXCL10 | GTGGCATTCAAGGAGTACCTC | GCCTTCGATTCTGGATTCAGACA |
| Human CSF-1 | cctgcgtccgaactttctat | cacatcttggctggagcat |
| Human CD86 | ctttccactcctggctgaga | tttcctctggttgccttgag |
| Human TNF-α | cccagggacctctctctaatc | tgaggtacaggccctctgat |
| Human IL-1β | gcatccagctacgaatctcc | gcatccagctacgaatctcc |
| Human IL-10 | ccacgctttctagctgttga | ctccgagacactggaaggtg |
| Human CD163 | AGCAGAGTTTGGTCAGGG | GGCTTTTTGTGGGGTTTTC |
| Human CD206 | CAGGTGTGGGCTCAGGTAGT | TGTGGTGAGCTGAAAGGTGA |
| Human IL-6 | AAATTCGGTACATCCTCGACGGCA | AGTGCCTCTTTGCTGCTTTCACAC |
| Human GAPDH | GAAGGTGAAGGTCGGAGTC | GAAGATGGTGATGGGATTTC |
| Mouse ALKBH5 | actcggcactttgcttcg | atcgcggtgcatctaatctt |
| Mouse JAK2 | GGTTCATTCAGCAGTTCAGTC | GCAGGGTCTCCAGGTTTATG |
| Mouse PD-L1 | GGCCGAGGGTTATCCAGAAG | AAACATCATTCGCTGTGGCG |
| Mouse CCL2 | ACTGAAGCCAGCTCTCTCTTCCTC | TTCCTTCTTGGGGTCAGCACAGAC |
| Mouse CXCL10 | GACGGTCCGCTGCAACTG | CTTCCCTATGGCCCTCATTCT |
| Mouse GAPDH | TGTGTCCGTCGTGGATCTGA | TTGCTGTTGAAGTCGCAGGAG |
| MeRIP–primer 1# | CCATTAGTAAACTGAAGAAAGCAG  G | TCGCTCGACAGCAAAAGTCAA |
| MeRIP–primer 2# | GTTTTCTGTGGCCTCAGATGTT | GCATAAATTCCGCTGGTGG |
