# Supplementary material for: ALKBH5 promotes non-small cell lung cancer progression and susceptibility to anti-PD-L1 therapy by modulating interactions between tumor and macrophages
Source: J Exp Clin Cancer Res. 2024 Jun 14;43:164. doi: 10.1186/s13046-024-03073-0 (PMC11177518; doi:10.1186/s13046-024-03073-0)
Supplement: Supplementary file 3 — Additional file 3: Table S3. Antibody sources and dilutions. [file 13046_2024_3073_MOESM3_ESM.doc]

**Table S3 Antibody sources and dilutions**

| **Western blotting** | **Company (Catalog#)** | **Dilution** |
| --- | --- | --- |
| anti-ALKBH5 | ABclonal Technology (A22137) | 1/1000 |
| anti-p-JAK2 | ABclonal Technology (AP0531) | 1/1000 |
| anti-JAK2 | Cell Signaling Technology (3230) | 1/1000 |
| anti-p-STAT3 | ABclonal Technology (AP0705) | 1/1000 |
| anti-PD-L1 | Proteintech (66248-1-Ig) | 1/2000 |
| anti-YTHDF2 | Proteintech (24744-1-AP) | 1/1000 |
| anti-GAPDH | Proteintech (60004-1-Ig) | 1/100000 |
| anti-rabbit | ABclonal Technology (AS014) | 1/5000 |
| anti-mouse | ABclonal Technology (AS003) | 1/5000 |

| **Immunofluorescence** | **Company (Catalog#)** | **Dilution** |
| --- | --- | --- |
| anti-p-STAT3 | Abcam (Ab76315) | 1/400 |
| anti-CD206 | Abcam (Ab64693) | 1/1000 |
| anti-PD-L1 | Proteintech (66248-1-1g) | 1/300 |
| anti-rabbit | Jackson ImmunoResearch | 1/400 |
| anti-mouse | Jackson ImmunoResearch | 1/400 |

| **Flow cytometry** | **Company (Catalog#)** |
| --- | --- |
| APC anti-CD11b | Biolegend (301310) |
| FITC anti-CD206 | Biolegend (321103) |
| PE anti-CD274 | Biolegend (329705) |

| **Immunohistochemistry** | **Company (Catalog#)** | **Dilution** |
| --- | --- | --- |
| anti-ALKBH5 | Proteintech (16837-1-AP) | 1/1500 |
| anti-CD8 | ABclonal Technology (A23305) | 1/500 |
| anti-CD68 | ABclonal Technology (A23205) | 1/2000 |
| anti-CD206 | ABclonal Technology (A21014) | 1/200 |
| Human anti-PD-L1 | ABclonal Technology (A11273) | 1/100 |
| Mouse anti-PD-L1 | Proteintech (66248-1-Ig) | 1/10000 |
| anti-JAK2 | ABclonal Technology (A11497) | 1/200 |
| anti-Ki-67 | ABclonal Technology (A21861) | 1/1000 |
| anti-F4/80 | Proteintech (28463-1-AP) | 1/2000 |
| anti-CD31 | Abcam (ab28364) | 1/50 |
