# Supplementary figures and images for: ALKBH5 promotes non-small cell lung cancer progression and susceptibility to anti-PD-L1 therapy by modulating interactions between tumor and macrophages
Source: J Exp Clin Cancer Res. 2024 Jun 14;43:164. doi: 10.1186/s13046-024-03073-0 (PMC11177518; doi:10.1186/s13046-024-03073-0)

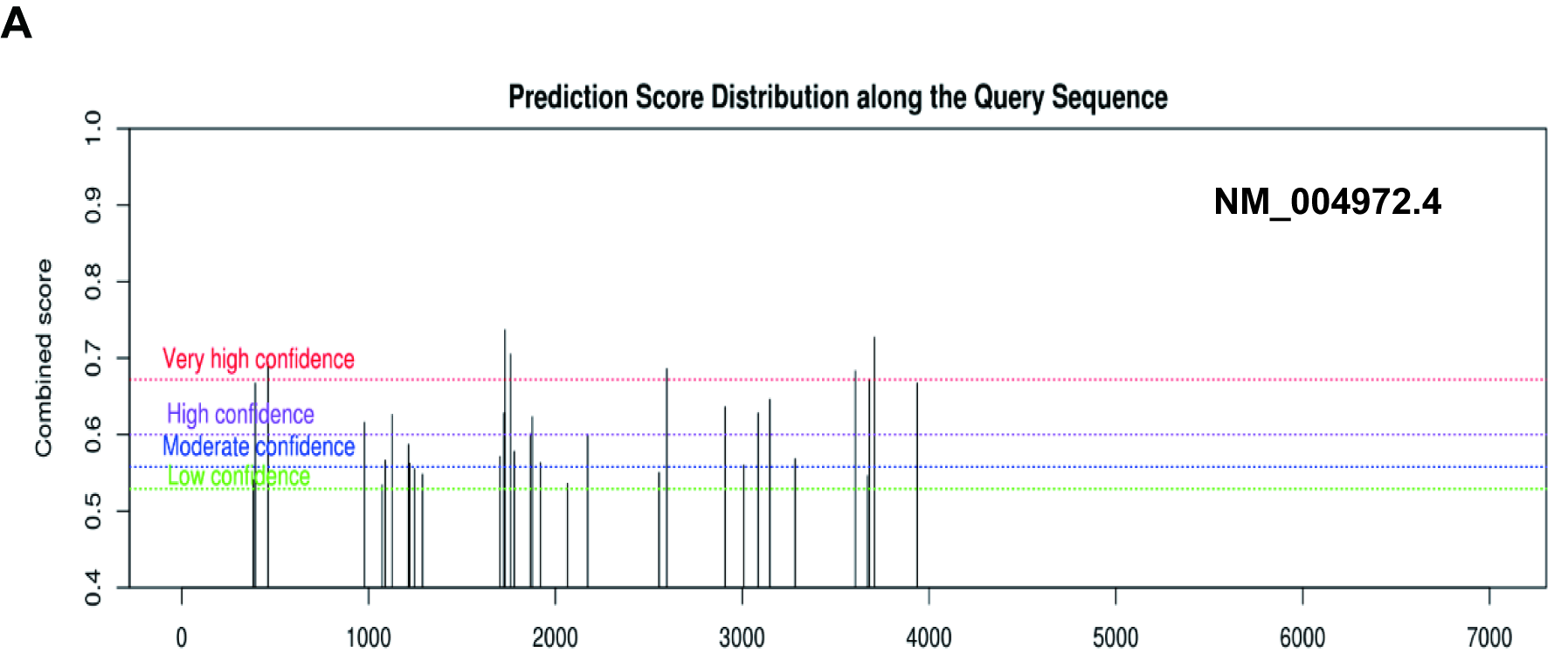

Supplement: Supplementary file 4 — Additional file 4: Figure S1. SRAMP predicted m6A modification sites on JAK2. A m6A modification sites predicted on the main JAK2 transcript based on online software SRAMP. [file 13046_2024_3073_MOESM4_ESM.tif]

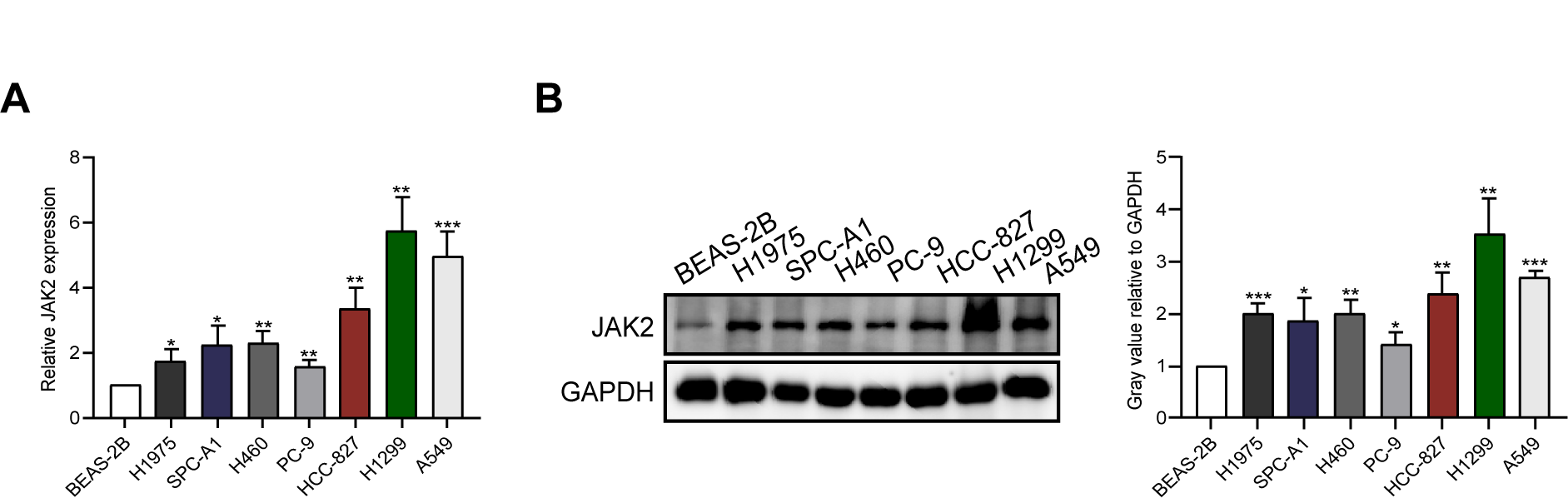

Supplement: Supplementary file 5 — Additional file 5: Figure S2. Basal expression level of JAK2 in normal bronchial epithelial cells and common NSCLC cells. A qRT-PCR analysis of JAK2 expression in A549, BEAS-2B, H460, H1299, H1975, HCC-827, PC-9, and SPC-A1 cells. B Western blot analysis of JAK2 expression in A549, BEAS-2B, H460, H1299, H1975, HCC-827, PC-9, and SPC-A1 cells. GAPDH was used as the loading control. *P < 0.05; **P < 0.01; ***P < 0.001. [file 13046_2024_3073_MOESM5_ESM.tif]

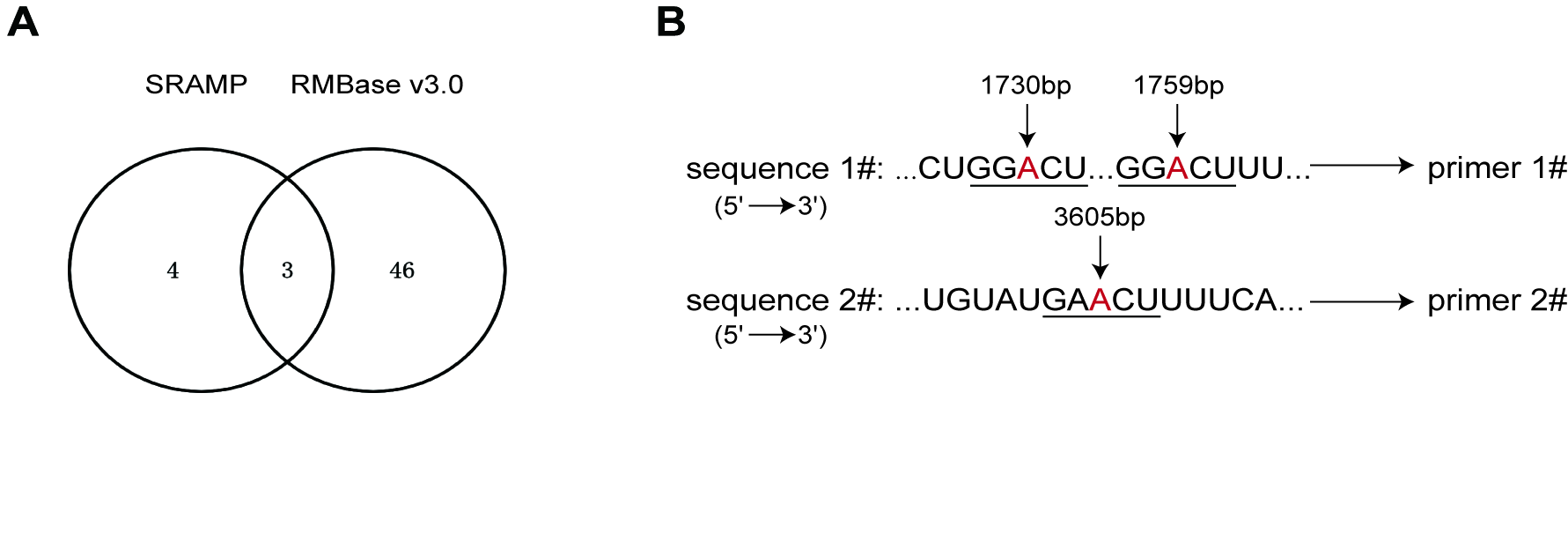

Supplement: Supplementary file 6 — Additional file 6: Figure S3. Construction of specific primers based on potential m6A modification sites on JAK2 transcript. A The online software SRAMP and RMBase V3.0 predicted 7 and 49 potential m6A modification sites on JAK2 transcript, respectively. B Three overlapping potential m6A modification sites on the JAK2 transcript were located at 1730 bp, 1759 bp, and 3605 bp on the JAK2 transcript from the 5’ end. Two pairs of specific primers were constructed to amplify the fragments near the sites (sequence 1 and sequence 2). [file 13046_2024_3073_MOESM6_ESM.tif]

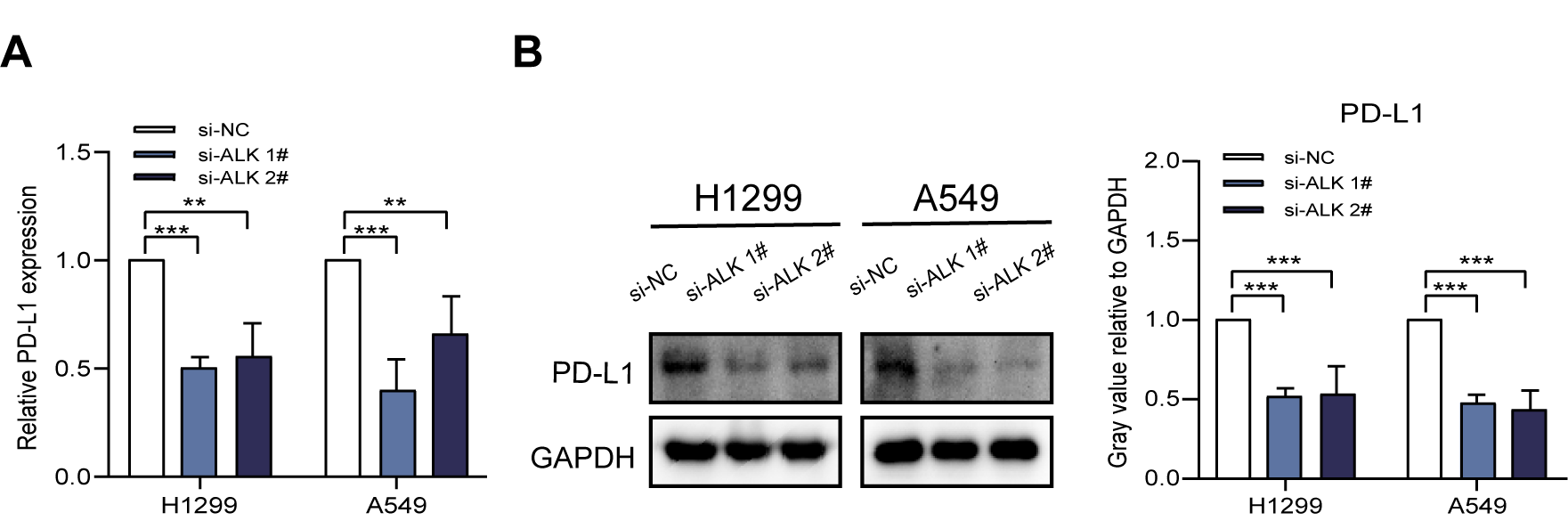

Supplement: Supplementary file 7 — Additional file 7: Figure S4. ALKBH5 induces PD-L1 expression in NSCLC cells. A qRT-PCR analysis of PD-L1 expression in A549 and H1299 cells transfected with ALKBH5 siRNA. B Western blot analysis of PD-L1 expression in A549 and H1299 cells transfected with ALKBH5 siRNA (loading control = GAPDH). **P < 0.01; ***P < 0.001. [file 13046_2024_3073_MOESM7_ESM.tif]

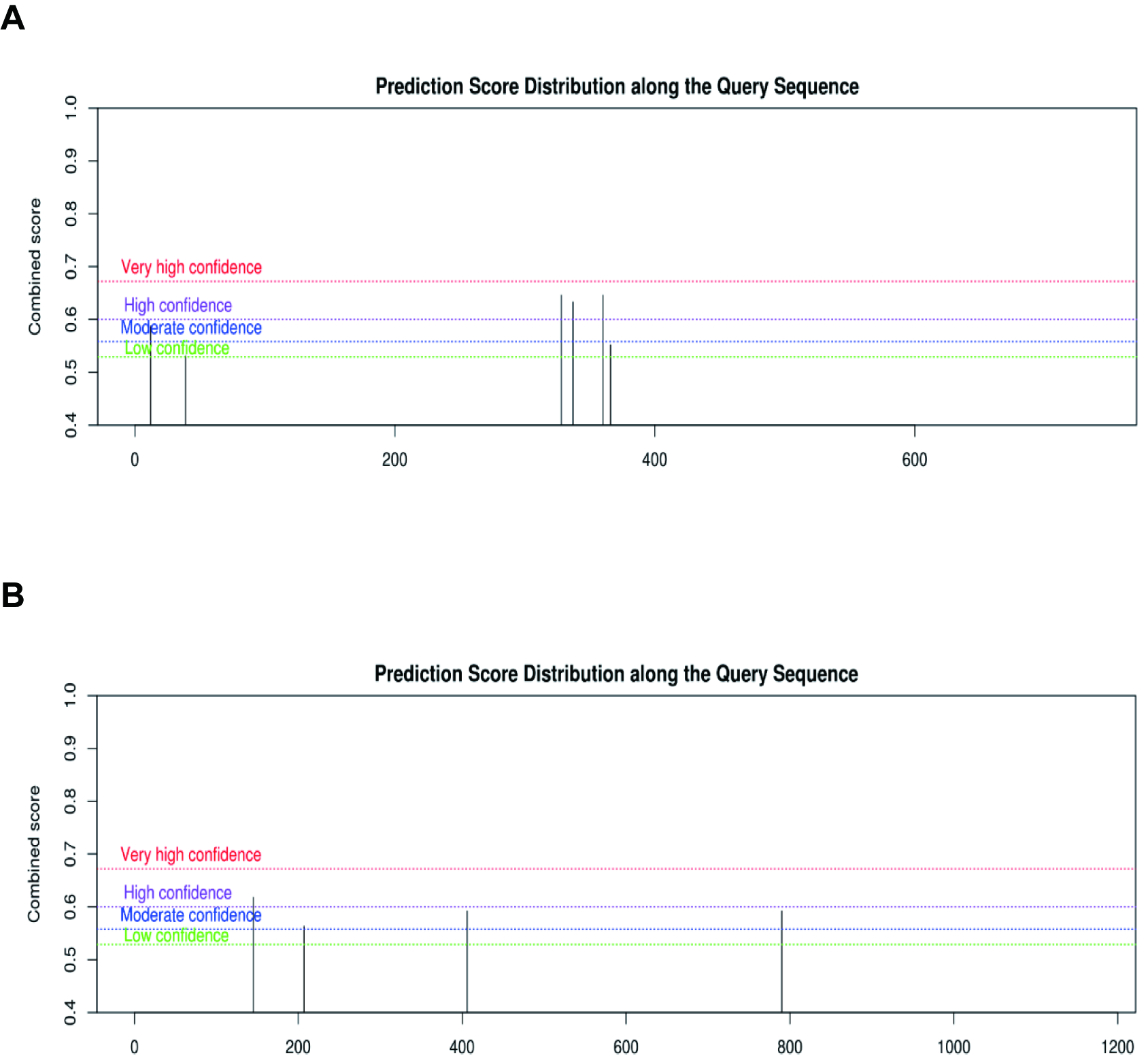

Supplement: Supplementary file 8 — Additional file 8: Figure S5. SRAMP predicted m6A modification sites on CCL2 and CXCL10. A m6A modification sites predicted on CCL2 based on online software SRAMP. B m6A modification sites predicted on CXCL10 based on online software SRAMP. [file 13046_2024_3073_MOESM8_ESM.tif]

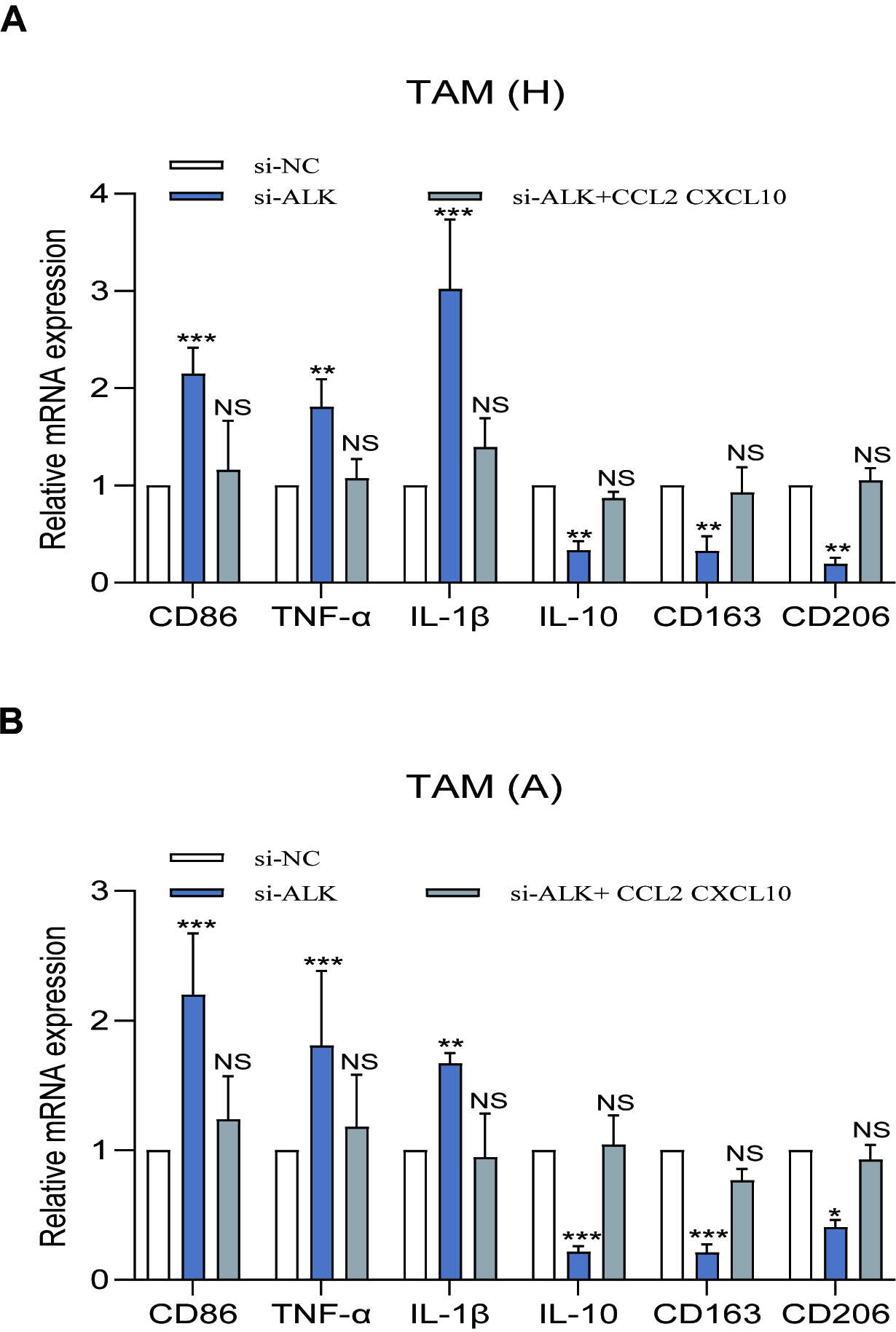

Supplement: Supplementary file 9 — Additional file 9: Figure S6 ALKBH5 promotes M2 macrophage polarization in NSCLC. A qRT-PCR analysis of the expression of M1 (CD86, IL-1β, and TNF-α) and M2 (CD163, CD206, and IL-10) polarization markers in PMA-stimulated THP-1 cells co-cultured with ALKBH5-knockdown H1299 cells treated with or without CCL2 (200 ng/mL) and CXCL10 (50 ng/mL) recombinant proteins. B qRT-PCR analysis of the expression of M1 (CD86, IL-1β, and TNF-α) and M2 (CD163, CD206, and IL-10) polarization markers in PMA-stimulated THP-1 cells co-cultured with ALKBH5-knockdown A549 cells treated with or without CCL2 (200 ng/mL) and CXCL10 (50 ng/mL) recombinant proteins. *P < 0.05; **P < 0.01; ***P < 0.001; NS, not significant. [file 13046_2024_3073_MOESM9_ESM.tif]
